# Semi-parametric estimates of population accuracy and bias of predictions of breeding values and future phenotypes using the LR method

**DOI:** 10.1186/s12711-018-0426-6

**Published:** 2018-11-06

**Authors:** Andres Legarra, Antonio Reverter

**Affiliations:** 1 INRA, UMR1388 GenPhySE, 31326 Castanet-Tolosan, France; 2 CSIRO Agriculture and Food, 306 Carmody Rd., St. Lucia, QLD 4067 Australia

## Abstract

**Background:**

Cross-validation tools are used increasingly to validate and compare genetic evaluation methods but analytical properties of cross-validation methods are rarely described. There is also a lack of cross-validation tools for complex problems such as prediction of indirect effects (e.g. maternal effects) or for breeding schemes with small progeny group sizes.

**Results:**

We derive the expected value of several quadratic forms by comparing genetic evaluations including “partial” and “whole” data. We propose statistics that compare genetic evaluations including “partial” and “whole” data based on differences in means, covariance, and correlation, and term the use of these statistics “method LR” (from linear regression). Contrary to common belief, the regression of true on estimated breeding values is (on expectation) lower than 1 for small or related validation sets, due to family structures. For validation sets that are sufficiently large, we show that these statistics yield estimators of bias, slope or dispersion, and population accuracy for estimated breeding values. Similar results hold for prediction of future phenotypes although we show that estimates of bias, slope or dispersion using prediction of future phenotypes are sensitive to incorrect heritabilities or precorrection for fixed effects. We present an example for a set of 2111 Brahman beef cattle for which, in repeated partitioning of the data into training and validation sets, there is very good agreement of statistics of method LR with prediction of future phenotypes.

**Conclusions:**

Analytical properties of cross-validation measures are presented. We present a new method named LR for cross-validation that is automatic, easy to use, and which yields the quantities of interest. The method compares predictions based on partial and whole data, which results in estimates of accuracy and biases. Prediction of observed records may yield biased results due to precorrection or use of incorrect heritabilities.

**Electronic supplementary material:**

The online version of this article (10.1186/s12711-018-0426-6) contains supplementary material, which is available to authorized users.

## Background

Models for genetic evaluation are an oversimplification of reality that usually holds only in the short run and in closely-related populations. Their properties are rarely well known, which can lead to unexpected results. For instance, initial applications of genomic predictions of breeding values (GEBV) in dairy cattle led to biases, with young “genomic” selected bulls with high GEBV being overpredicted, as verified by posterior progeny testing [1–3]. As a result, further use of GEBV in the dairy industry required extensive cross-validation and a more formal analytical framework [4–6].

The introduction of new methods for genetic or genomic evaluation raises the question of model choice (comparing across models) and model quality (features of a particular model). Thus, we need tools to rank, understand and quantify the behavior of prediction models in an “animal breeding” context. The need for these tools has dramatically increased with the implementation of genomic selection, given its built-in encouragement to take riskier decisions such as selection of unproven young candidates, in particular in dairy cattle. The method that is most commonly used to check genomic predictions is some form of cross-validation, a test that was rarely used in pedigree-based genetic evaluation studies, which relied primarily on progeny testing (but see [7, 8]). In genomic prediction, cross-validation studies are indeed the norm [4, 9, 10].

Cross-validation tests rely on either one of two approaches: (1) comparing (G)EBV or predicted phenotypes to (pre-corrected) observed phenotypes, deregressed proofs, or yield deviations [9]; or (2) comparing (G)EBV to highly accurate EBV from progeny testing. Another approach, which is in between the two above approaches, is based on daughter yield deviations (DYD; [6]), which are close to highly accurate EBV if heritability is high and the number of daughters is large. Cross-validation is very useful but there are some concerns about the quality or adequacy of these approaches for several reasons, including: (a) the need to pre-correct phenotypes; (b) the growing difficulty to obtain unbiased estimates of DYD with the increasing use of non-progeny tested bulls selected based on GEBV; and (c) their inadequacy for indirect predictions such as those of maternal effects, for which there is no direct observation related to the animal. Apparent contradictions exist, such as lower accuracy of GEBV than that of pedigree EBV [5, 11], or accuracies higher than 1 for lowly heritable traits. For a detailed review of cross-validation in animal breeding and its metrics, we refer the reader to our review [12].

Cross-validation is a good tool but has some limitations as discussed above. Thus, there is an increasing need for a simple general tool that can be used in several complex scenarios, including for traits with a low heritability (e.g. reproductive and fitness traits), for indirectly observed traits (random regression coefficients, maternal effects, GxE interactions), and with limited size of progeny test groups (e.g., pigs). Here, we propose to complement cross-validation approaches with semiparametric procedures based on the classical theory of genetic evaluation.

Semiparametric procedures based on the mixed model equations are appealing because they combine theory, which we know is approximately and/or asymptotically correct, with model-free evidence from data. In the 1990’s, there was some effort to develop such procedures [13]. Reverter et al. [14] showed that the amount of change in EBV from one genetic evaluation to the next (i.e., with the arrival of “new” data) was predictable. In parallel, bias in across-country predictions [15, 16] led to the introduction of the Interbull tests [17], which draw on a similar idea. This family of methods has been used to check unbiasedness of predictions and, in the case of the Interbull tests, relies heavily on the availability of progeny tests based on large numbers of daughters.

In this work, we draw on analytical results from [14] and present theoretical features of semi-parametric procedures, namely method LR (from “linear regression”). These procedures are a series of statistics, which describe the change of predictions from “old” to “recent” evaluations that can be used to compute and compare population accuracies and biases of (genomic) predictions. We also explore analytical properties of the ability to predict future phenotypes, sometimes called “predictivity”. Then, we illustrate the method with deterministic results for simple designs and for experimental beef cattle data.

This work proposes estimates of the “population” accuracy, which is the correlation between true (TBV) and estimated breeding values (EBV) across individuals in a population. Population accuracy is relevant to compare the predictive ability of models and to maximize genetic progress. This work does *not* propose methods to estimate individual accuracies, which are a measure of the risk when choosing a particular animal for breeding [18].

## Methods: analytical developments

We propose to test the quality of evaluation methods using cross-validation tests based on successive EBV of a set of “focal” individuals (a validation cohort). These “focal” individuals can be the whole population [14, 19] or a set of “focal” individuals of interest, such as “genomic” candidates for selection [6].

We will use the convention that $var\left( {\mathbf{x}} \right)$ refers to a scalar, the variance of a random element from a single realization of random vector ${\mathbf{x}}$ (in other words, $var\left( {\mathbf{x}} \right) = \frac{1}{n}\mathop \sum \nolimits_{i} x_{i}^{2} - \left( {\frac{1}{n}\mathop \sum \nolimits_{i} x_{i} } \right)^{2}$ where *n* is the size of ${\mathbf{x}}$), whereas $Var\left( {\mathbf{x}} \right)$ refers to the variance–covariance matrix of elements in ${\mathbf{x}}$ during conceptual repetitions. We use a similar convention for $cov\left( {{\mathbf{x}},{\mathbf{y}}} \right)$ and $r\left( {{\mathbf{x}},{\mathbf{y}}} \right)$, which are scalars that represent the covariance and correlation across elements in ${\mathbf{x}}$ and ${\mathbf{y}}$, whereas $Cov\left( {{\mathbf{x}},{\mathbf{y}}} \right)$ is a matrix.

### Definition of population accuracy, bias, and dispersion

Let $u$ be the true breeding value (TBV) and $\hat{u}$ an estimated breeding value (EBV) of a single individual. The classical definition of accuracy is the correlation $r\left( {u,\hat{u}} \right)$ for one individual across conceptual repeated sampling [20], which is a measure of the expected magnitude of the change in EBV with increasing information. Accuracies are also used to forecast genetic progress in a selection scheme [18, 21, 22]. This use applies to large unrelated populations, and made sense at the time of selection-index based selection (e.g. selecting boars based on family information). However, for the joint evaluation of all animals, the relevant measure according to Bijma [18] is “the correlation between true i.e. TBV and EBV in the candidates for selection, which is a property of a population, not of an individual”. This “population accuracy” (we will use this term hereafter) is the correlation $r\left( {{\mathbf{u}},{\hat{\mathbf{u}}}} \right) = cov\left( {{\mathbf{u}},{\hat{\mathbf{u}}}} \right)/\sqrt {var\left( {{\hat{\mathbf{u}}}} \right)var\left( {\mathbf{u}} \right)}$ across a series of individuals.

Accordingly, bias is defined as the difference of means ${\bar{\mathbf{u}}} - {\bar{\hat{\mathbf{u}}}}$ and dispersion as the slope of the regression of ${\mathbf{u}}$ on ${\hat{\mathbf{u}}}$: $cov\left( {{\mathbf{u}},{\hat{\mathbf{u}}}} \right)/var\left( {{\hat{\mathbf{u}}}} \right)$. Indeed, in practice, proxies to these empirical measures are used in cross-validation studies. In other words, accuracy measures the ability to rank individuals within the focal set of individuals, taking the possible relatedness within the sample into account [23, 24], as well as the buildup of the Bulmer effect that reduces genetic variance and makes evaluation more difficult [18, 25].

Note that the three quantities accuracy, bias, and dispersion are defined as *scalars*, i.e.

$$\begin{aligned} var\left( {{\hat{\mathbf{u}}}} \right) & = \frac{1}{n} \mathop \sum \limits_{i} \hat{u}_{i}^{2} - \left( {\frac{1}{n} \mathop \sum \limits_{i} \hat{u}_{i} } \right)^{2} \\ & \ne Var\left( {{\hat{\mathbf{u}}}} \right) = \left( {\begin{array}{*{20}c} {Var\left( {\hat{u}_{1} } \right)} & {Cov\left( {\hat{u}_{1} ,\hat{u}_{2} } \right)} & \\ & {Var\left( {\hat{u}_{2} } \right)} & \\ & & \ldots \\ \end{array} } \right), \\ \end{aligned}$$

and have distributions, i.e. over conceptual repetitions $r\left( {{\mathbf{u}},{\hat{\mathbf{u}}}} \right)$ have themselves a mean and a variance.

We also use indicators of (self-)relationships and of genetic variances within the sample. If the relationship matrix across focal individuals is ${\mathbf{K}}$, then we use $\overline{{diag\left( {\mathbf{K}} \right)}} - {\bar{\mathbf{K}}} = 1 + \bar{F} - 2\bar{f}$ where $F$ is the inbreeding coefficient and $2f$ is the relationship between individuals ($f$ can be understood as coancestry), and the bar operators imply averages, i.e. ${\bar{\mathbf{X}}}$ is the average across elements of ${\mathbf{X}}$. The statistic $\overline{{diag\left( {\mathbf{K}} \right)}} - {\bar{\mathbf{K}}}$ was used by [26] to describe the decrease in genetic variance due to relationships in a related but unselected population. For selected populations, even of infinite size, there is a further decrease in genetic variance due to the Bulmer effect [18, 27], and we will use $\sigma_{u,\infty }^{2} = \left( {1 - k} \right)\sigma_{u }^{2}$ where $k$ is the reduction due to selection and $\sigma_{u,\infty }^{2}$ is the genetic variance at equilibrium in a population under selection. The equivalence between Henderson’s [28] results for the decrease in genetic variance in a selected population and $\sigma_{u,\infty }^{2} = \left( {1 - k} \right)\sigma_{u }^{2}$ was shown (in simplified settings) by [27, 29].

### Statistics to test the quality of evaluation methods in brief

Consider successive evaluations with “partial” and “whole” data (${\hat{\mathbf{u}}}_{p}$ and ${\hat{\mathbf{u}}}_{w}$, respectively), which is based on the use of “old” ($p$) and “recent + old” ($w$ from “whole”) phenotype data, respectively. Note that in the following, ${\hat{\mathbf{u}}}_{p}$ and ${\hat{\mathbf{u}}}_{w}$ have the same dimension and may be a subset of “focal” individuals (e.g. the young candidates for selection) or the number of animals in the entire dataset (i.e., in the relationship matrix). In general, the breeder is concerned with the population accuracy of candidates for selection, because higher population accuracy of selection candidates implies greater genetic progress. Typically, focal individuals have no phenotype (or offspring phenotyped) in $p$ but have phenotype (or offspring phenotyped) in $w$, but this is not a requirement for the proposed method. Reverter et al. [14] described the amount of change that is expected in consecutive genetic evaluations of individuals as a function of their respective accuracies, and they proposed statistics to check biases in genetic evaluations. The proposed criteria were very beneficial because (1) they do not require knowledge of the TBV, only the EBV from successive evaluations, and (2) they do not require knowledge of adjustment factors to pre-correct phenotypes.

In general, assumptions are: $Cov\left( {{\hat{\mathbf{u}}}_{w} ,{\hat{\mathbf{u}}}_{p} } \right) = Var\left( {{\hat{\mathbf{u}}}_{p} } \right)$, $E\left( {{\hat{\mathbf{u}}}_{p} } \right) = E\left( {{\hat{\mathbf{u}}}_{w} } \right) = E\left( {\mathbf{u}} \right)$ and $Cov\left( {{\mathbf{u}} - {\hat{\mathbf{u}}}_{p} ,{\hat{\mathbf{u}}}_{p} } \right) = Cov\left( {{\mathbf{u}} - {\hat{\mathbf{u}}}_{w} ,{\hat{\mathbf{u}}}_{w} } \right) = 0$. Henderson [28] proved that $Cov\left( {{\mathbf{u}},{\hat{\mathbf{u}}}} \right) = Var\left( {{\hat{\mathbf{u}}}} \right)$ even in the presence of selection, which when coupled with the results in [14] yields $Cov\left( {{\hat{\mathbf{u}}}_{w} ,{\hat{\mathbf{u}}}_{p} } \right) = Var\left( {{\hat{\mathbf{u}}}_{p} } \right)$. Intuitively, this holds if “old” errors in prediction ($u - \hat{u}_{p}$) are uncorrelated with “new” information, which in turn holds if the model takes selection correctly into account. Another assumption, which will be shown later in this paper, is that the set of focal individuals is sufficiently large and “diverse” (for instance, there are several full-sib families and not just one). The derivations of Reverter et al. [14] referred to the individual case (e.g. $r\left( {u,\hat{u}} \right)$) and not to *sets* of individuals (e.g. $r\left( {{\mathbf{u}},{\hat{\mathbf{u}}}} \right)$) that are used for cross-validation. We extend their results as shown below, which leads to the following main results.

1. The statistic $\mu_{wp} = \overline{{{\hat{\mathbf{u}}}_{p} }} - \overline{{{\hat{\mathbf{u}}}_{w} }}$, has an expected value of 0 if the evaluation is unbiased.
2. The regression of EBV obtained with “whole” ($w$) data on EBV estimated with “partial” ($p$) data $b_{w,p} = \frac{{cov\left( {{\hat{\mathbf{u}}}_{w} ,{\hat{\mathbf{u}}}_{p} } \right)}}{{var\left( {{\hat{\mathbf{u}}}_{p} } \right)}}$ has an expectation, $E\left( {b_{w,p} } \right) = 1$ if there is no over/under dispersion.
3. The correlation of EBV based on partial and whole data, $\rho_{p,w} = \frac{{cov\left( {{\hat{\mathbf{u}}}_{p} ,{\hat{\mathbf{u}}}_{w} } \right)}}{{\sqrt {var\left( {{\hat{\mathbf{u}}}_{w} } \right)var\left( {{\hat{\mathbf{u}}}_{p} } \right)} }}$, is a function of their respective accuracies ($acc$), with an expected value $E\left( {\rho_{w,p} } \right) \approx \frac{{acc_{p} }}{{acc_{w} }}$, where $acc$ is the population accuracy (correlation between TBV and EBV across animals).
4. The covariance of EBV based on partial and whole data is a function of the squared accuracy (reliability) of the partial EBV, $\rho_{{Cov_{w,p} }}^{2} = \frac{{cov\left( {{\hat{\mathbf{u}}}_{w} ,{\hat{\mathbf{u}}}_{p} } \right)}}{{\left( {1 + \bar{F} - 2\bar{f}} \right)\sigma_{u,\infty }^{2} }}$, $E\left( {\rho_{{Cov_{w,p} }}^{2} } \right) \approx acc_{p}^{2}$.
5. The slope of the regression of EBV based on partial on EBV based on whole data, $b_{p,w} = \frac{{cov\left( {{\hat{\mathbf{u}}}_{w} ,{\hat{\mathbf{u}}}_{p} } \right)}}{{var\left( {{\hat{\mathbf{u}}}_{w} } \right)}}$ is, on expectation, a function of the respective accuracies $E\left( {b_{p,w} } \right) = \frac{{acc_{p}^{2} }}{{acc_{w}^{2} }}$ that is, the expectation of the slope is proportional to the relative increase in average reliabilities from EBV based on partial to EBV based on whole data.

### Proofs of the adequacy of the statistics

In the following, we prove that the statistics described above are related to bias, slope and accuracies. We make repeated use of the following results for biquadratic forms [30]: consider random vectors ${\mathbf{x}}_{1}$, ${\mathbf{x}}_{2}$ such that $$E\left( {\begin{array}{*{20}c} {{\mathbf{x}}_{1} } \\ {{\mathbf{x}}_{2} } \\ \end{array} } \right) = \left( {\begin{array}{*{20}c} {{\varvec{\upmu}}_{1} } \\ {{\varvec{\upmu}}_{2} } \\ \end{array} } \right); Var\left( {\begin{array}{*{20}c} {{\mathbf{x}}_{1} } \\ {{\mathbf{x}}_{2} } \\ \end{array} } \right) = \left( {\begin{array}{*{20}c} {{\mathbf{V}}_{11} } & {{\mathbf{V}}_{21} } \\ {{\mathbf{V}}_{12} } & {{\mathbf{V}}_{22} } \\ \end{array} } \right)$$. The expectation of the quadratic form ${\mathbf{x}}_{1}^{{\prime }} {\mathbf{A}}_{12} {\mathbf{x}}_{2}$ is $E\left( {{\mathbf{x}}_{1}^{'} {\mathbf{A}}_{12} {\mathbf{x}}_{2} } \right) = tr\left( {{\mathbf{A}}_{12} {\mathbf{V}}_{12} } \right) + {\varvec{\upmu}}_{1}^{'} {\mathbf{A}}_{12} {\varvec{\upmu}}_{2}$. Empirical (co)variances (scalars) are quadratic forms, for instance $cov\left( {{\hat{\mathbf{u}}}_{w} ,{\hat{\mathbf{u}}}_{p} } \right) = \frac{1}{n}\left( {{\hat{\mathbf{u}}}_{p} - {\bar{\hat{\mathbf{u}}}}_{p} } \right)^{'} \left( {{\hat{\mathbf{u}}}_{w} - {\bar{\hat{\mathbf{u}}}}_{w} } \right) = \frac{1}{n}{\hat{\mathbf{u}}}_{p}^{'} {\mathbf{S}{\hat{\mathbf{u}}}}_{w}$ where ${\mathbf{S}} = {\mathbf{I}} - \frac{1}{n}{\mathbf{J}}$ is the centering matrix [31].

Considering our problem, we make the hypothesis that the two genetic evaluations (e.g. males *before* (“partial”) and *after* (“whole”) progeny testing) have different means:

$$E\left( {\begin{array}{*{20}c} {{\hat{\mathbf{u}}}_{p} } \\ {{\hat{\mathbf{u}}}_{w} } \\ \end{array} } \right) = \left( {\begin{array}{*{20}c} {\mathbf{1}\mu_{p} } \\ {\mathbf{1}\mu_{w} } \\ \end{array} } \right).$$

Note that the meaning of the “expected mean of EBV ${\hat{\mathbf{u}}}$” is unclear under selection. For instance, the last generation is expected to have means higher than 0, but these means will differ for males (heavily selected) and females (less selected). We will assume that the focal individuals include sets of animals that are comparable, i.e. under repeated sampling they have the same *average* genetic level. For instance, if 1% of the elite females and 10% of the elite bulls are selected, offspring from these animals should have on average the same genetic level across conceptual repetitions of the breeding scheme; the actual animals that are selected will differ but the genetic gain will on average be the same. We also assume (as commonly done) that, because of selection, $Var\left( {\mathbf{u}} \right) = {\mathbf{G}} = {\mathbf{K}}\sigma_{u,\infty }^{2}$ (${\mathbf{K}}$ is a relationship matrix) instead of the non-selection case, $Var\left( {\mathbf{u}} \right) = {\mathbf{G}} = {\mathbf{K}}\sigma_{u}^{2}$. This implies that Henderson’s [28] description of decrease in variance due to the Bulmer effect is correct and that it can be summarized by a single parameter $\sigma_{u,\infty }^{2}$ [18, 25, 27].

Following classical notation: $Var\left( {\mathbf{u}} \right) = {\mathbf{G}} = {\mathbf{K}}\sigma_{u}^{2}$ and $Var\left( {{\hat{\mathbf{u}}} - {\mathbf{u}}} \right) = {\mathbf{C}}^{uu}$, and the matrix of prediction error variances and covariances (PEV and PEC) can be described as [14, 28].

$$Var\left( {\begin{array}{*{20}c} {{\hat{\mathbf{u}}}_{p} } \\ {{\hat{\mathbf{u}}}_{w} } \\ {\mathbf{u}} \\ \end{array} } \right) = \left( {\begin{array}{*{20}c} {{\mathbf{G}} - {\mathbf{C}}_{p}^{uu} } & {{\mathbf{G}} - {\mathbf{C}}_{p}^{uu} } & {{\mathbf{G}} - {\mathbf{C}}_{p}^{uu} } \\ {{\mathbf{G}} - {\mathbf{C}}_{p}^{uu} } & {{\mathbf{G}} - {\mathbf{C}}_{w}^{uu} } & {{\mathbf{G}} - {\mathbf{C}}_{w}^{uu} } \\ {{\mathbf{G}} - {\mathbf{C}}_{p}^{uu} } & {{\mathbf{G}} - {\mathbf{C}}_{w}^{uu} } & {\mathbf{G}} \\ \end{array} } \right),$$

where ${\mathbf{C}}_{p}^{uu}$ and ${\mathbf{C}}_{w}^{uu}$ are matrices of PEV and PEC for the partial and whole analysis, respectively. These expressions assume that genetic evaluation deals correctly with the decrease in genetic variance due to selection [28, 32] in which case $Var\left( {{\hat{\mathbf{u}}}_{p} } \right) = Cov\left( {{\hat{\mathbf{u}}}_{p} ,{\hat{\mathbf{u}}}_{w} } \right)$. From here, we derive expectations of several possible quadratic forms that are combined to produce estimators of bias, dispersion and accuracy. In principle, genetic evaluation does not need to be based on mixed models (statistics can be computed regardless of the procedure), but our results only hold if the variances and covariances of estimators and true values are as described above. Average inbreeding and relationships in ${\mathbf{K}}$ are also needed. Ideally, the evaluation is based on conditional means such that the properties described before hold. Precision of the estimators of accuracy and bias depends on the distributional properties of the EBV and TBV, which can be derived when assuming multivariate normality but we have not attempted to do so herein.

#### Averages of estimated breeding values to estimate bias

It is straightforward to show that $E\left( {\frac{{\mathbf{1}^{{\prime }} {\hat{\mathbf{u}}}_{p} }}{n}} \right) = \mu_{p}$, $E\left( {\frac{{\mathbf{1}^{{\prime }} {\hat{\mathbf{u}}}_{w} }}{n}} \right) = \mu_{w}$. Thus, $\mu_{wp} = \overline{{{\hat{\mathbf{u}}}_{p} }} - \overline{{{\hat{\mathbf{u}}}_{w} }}$ is a direct measure of bias.

#### Quadratic forms of estimated breeding values

For the method R of covariance estimation, it is recommended that the dispersion (relationship) matrix ${\mathbf{K}}$ ($Var\left( {\mathbf{u}} \right) = {\mathbf{G}} = {\mathbf{K}}\sigma_{u}^{2} )$ is included in the quadratic forms, especially in the presence of selection [33]:

$$\begin{aligned} E\left( {{\hat{\mathbf{u}}}_{p}^{{\prime }} {\mathbf{K}}^{ - 1} {\hat{\mathbf{u}}}_{w} } \right) & = tr\left( {{\mathbf{K}}^{ - 1} \left( {{\mathbf{K}}\sigma_{u}^{2} - {\mathbf{C}}_{p}^{uu} } \right)} \right) + \mu_{p} \mathbf{1}^{{\prime }} {\mathbf{G}}^{ - 1} \mathbf{1}\mu_{w} \\ & = tr\left( {\mathbf{I}} \right)\sigma_{u}^{2} - tr\left( {{\mathbf{K}}^{ - 1} {\mathbf{C}}_{p}^{uu} } \right) + \mu_{p} \mathbf{1}^{{\prime }} {\mathbf{K}}^{ - 1} \mathbf{1}\mu_{w} . \\ \end{aligned}$$

However, these weighted quadratic forms lead to estimators that are difficult to understand. Hence, in the following, we will use “unweighted” quadratic forms.

The quadratic form using not-centered ${\hat{\mathbf{u}}}_{p}$, ${\hat{\mathbf{u}}}_{w}$ has expectation:

$$\begin{aligned} E\left( {{\hat{\mathbf{u}}}_{p}^{{\prime }} {\hat{\mathbf{u}}}_{w} } \right) & = tr\left( {{\mathbf{G}} - {\mathbf{C}}_{p}^{uu} } \right) + \mu_{p} \mathbf{1}^{{\prime }} \mathbf{1}\mu_{w} \\ & = n\left( {1 + \bar{F}} \right)\sigma_{u,\infty }^{2} - n\overline{{PEV_{p} }} + n\mu_{w} \mu_{p} , \\ \end{aligned}$$

where $n$ is the number of individuals, $1 + \bar{F}$ is the average self-relationship, $\overline{{PEV_{p} }} = \overline{{diag\left( {{\mathbf{C}}_{p}^{uu} } \right)}}$ is the average prediction error variance and $\sigma_{u,\infty }^{2}$ is the genetic variance. It is worth noting that the classical definition of individual accuracy is based on $acc_{i}^{2} = \frac{{\left( {1 + F_{i} } \right)\sigma_{u}^{2} + PEVp_{i} }}{{\left( {1 + F_{i} } \right)\sigma_{u}^{2} }}$ [20]. Thus, the expression above for $E\left( {{\hat{\mathbf{u}}}_{p}^{{\prime }} {\hat{\mathbf{u}}}_{w} } \right)$ is a function of individual expected average reliabilities $acc_{i}^{2}$, but also of means $\mu_{w}$, $\mu_{p}$.

To remove dependence of the quadratic form above on means, it makes sense to use centered ${\hat{\mathbf{u}}}_{w}$ and ${\hat{\mathbf{u}}}_{p}$:

$$E\left( {\left( {{\hat{\mathbf{u}}}_{p} - {\bar{\hat{\mathbf{u}}}}_{p} } \right)^{'} \left( {{\hat{\mathbf{u}}}_{w} - {\bar{\hat{\mathbf{u}}}}_{w} } \right)} \right) = tr\left( {{\mathbf{S}}^{{\prime }} {\mathbf{S}}\left( {{\mathbf{G}} - {\mathbf{C}}_{p}^{uu} } \right)} \right) + \mu_{p} \mathbf{1}^{{\prime }} {\mathbf{S}}^{{\prime }} {\mathbf{S}}\mathbf{1}\mu_{w} ,$$

where ${\mathbf{S}} = {\mathbf{I}} - \frac{1}{n}{\mathbf{J}}$ is the centering matrix [31]. By its properties, ${\mathbf{S1}} = \mathbf{0}$ and ${\mathbf{S}}^{{\prime }} {\mathbf{S}} = {\mathbf{S}}^{{\prime }} = {\mathbf{S}}$, and therefore

$$\begin{aligned} E\left( {\frac{1}{n}\left( {{\hat{\mathbf{u}}}_{p} - {\bar{\hat{\mathbf{u}}}}_{p} } \right)^{'} \left( {{\hat{\mathbf{u}}}_{w} - {\bar{\hat{\mathbf{u}}}}_{w} } \right)} \right) & = \frac{1}{n}tr\left( {{\mathbf{SG}} - {\mathbf{SC}}_{p}^{uu} } \right) \\ & = \overline{{diag\left( {\mathbf{G}} \right)}} - {\bar{\mathbf{G}}} - \left( {\overline{{diag\left( {{\mathbf{C}}_{p}^{uu} } \right)}} - \overline{{\left( {{\mathbf{C}}_{p}^{uu} } \right)}} } \right) \\ & = \left( {1 + \bar{F} - 2\bar{f}} \right)\sigma_{u,\infty }^{2} - \left( {\overline{{PEV_{p} }} - \overline{{PEC_{p} }} } \right), \\ \end{aligned}$$

is a function of the average self-relationships $1 + F$ minus the average relationship between individuals, $2f$, and PEV minus PEC. Inclusion of relationships between individuals results in the corresponding reduction in genetic variance due to inbreeding to be accounted for, i.e., if as usual $F \approx f$, then $\left( {1 + \bar{F} - 2\bar{f}} \right)\sigma_{u}^{2} = \left( {1 - \bar{F}} \right)\sigma_{u}^{2}$, which shows the decrease in genetic variance [26, 34, 35]. Similarly, $\overline{{PEV_{p} }} - \overline{{PEC_{p} }}$ considers the fact that estimates of $\hat{u}$ are correlated across individuals (the so-called “co-reliabilities” [25]), showing that there is little value in having high individual accuracy if predictors are correlated across individuals.

The remaining quadratic forms needed for our developments are:

$$\begin{aligned} & E\left( {\frac{1}{n}\left( {{\hat{\mathbf{u}}}_{p} - {\bar{\hat{\mathbf{u}}}}_{p} } \right)^{'} \left( {{\hat{\mathbf{u}}}_{p} - {\bar{\hat{\mathbf{u}}}}_{p} } \right)} \right) \\ & \quad = \left( {1 + \bar{F} - 2\bar{f}} \right)\sigma_{u,\infty }^{2} - \left( {\overline{{PEV_{p} }} - \overline{{PEC_{p} }} } \right), \\ \end{aligned}$$

and

$$\begin{aligned} & E\left( {\frac{1}{n}\left( {{\hat{\mathbf{u}}}_{w} - {\bar{\hat{\mathbf{u}}}}_{w} } \right)^{'} \left( {{\hat{\mathbf{u}}}_{w} - {\bar{\hat{\mathbf{u}}}}_{w} } \right)} \right) \\ & \quad = \left( {1 + \bar{F} - 2\bar{f}} \right)\sigma_{u,\infty }^{2} - \left( {\overline{{PEV_{w} }} - \overline{{PEC_{w} }} } \right). \\ \end{aligned}$$

In the remainder of this paper, we assume that the expectation of a ratio of quadratic forms is equal to the ratio of the expectations. The “Appendix” shows that this holds when the number of individuals included in the statistics is large (several hundred or more) or when they are not structured into very large sibships. Otherwise, as shown in the “Appendix”, both the true regression coefficient $b = cov\left( {{\hat{\mathbf{u}}}_{p} ,{\mathbf{u}}} \right)/var\left( {{\hat{\mathbf{u}}}_{p} } \right)$ and its estimator $\hat{b} = cov\left( {{\hat{\mathbf{u}}}_{p} ,{\hat{\mathbf{u}}}_{w} } \right)/var\left( {{\hat{\mathbf{u}}}_{p} } \right)$ have an expectation less than 1, even when the model is perfect and the EBV have the right dispersion.

#### Quadratic forms of estimated and true breeding values

Here, we give an alternative definition of the population accuracy, i.e. the expected correlation of EBV and TBV in a sample, as a ratio of quadratic forms:

$$acc_{p} = E\left( {\rho_{T,p} } \right) = {\text{E}}\left( {\frac{{ \left( {{\hat{\mathbf{u}}}_{p} - {\bar{\hat{\mathbf{u}}}}_{p} } \right)^{ '} \left( {{\mathbf{u}} - {\bar{\mathbf{u}}}} \right)}}{{\sqrt { \left( {{\mathbf{u}} - {\bar{\mathbf{u}}}} \right)^{{\prime }} \left( {{\mathbf{u}} - {\bar{\mathbf{u}}}} \right) \left( {{\hat{\mathbf{u}}}_{p} - {\bar{\hat{\mathbf{u}}}}_{p} } \right)^{{\prime }} \left( {{\hat{\mathbf{u}}}_{p} - {\bar{\hat{\mathbf{u}}}}_{p} } \right)} }}} \right).$$

Using

$$\begin{aligned} E\left( {\left( {{\hat{\mathbf{u}}}_{p} - {\bar{\hat{\mathbf{u}}}}_{p} } \right)^{{\prime }} \left( {{\mathbf{u}} - {\bar{\mathbf{u}}}} \right)} \right) = tr\left( {{\mathbf{S}}\left( {{\mathbf{G}} - {\mathbf{C}}_{p}^{uu} } \right)} \right) \hfill \\ = \left( {1 + \bar{F} - 2\bar{f}} \right)\sigma_{u,\infty }^{2} - \left( {\overline{{PEV_{p} }} - \overline{{PEC_{p} }} } \right), \hfill \\ \end{aligned}$$

$$E\left( {\left( {{\mathbf{u}} - {\bar{\mathbf{u}}}} \right)^{{\prime }} \left( {{\mathbf{u}} - {\bar{\mathbf{u}}}} \right)} \right) = tr\left( {{\mathbf{SG}}} \right) = \left( {1 + \bar{F} - 2\bar{f}} \right)\sigma_{u,\infty }^{2} ,$$

and $E\left[ {\left( {{\mathbf{u}} - {\bar{\mathbf{u}}}} \right)^{{\prime }} \left( {{\hat{\mathbf{u}}}_{p} - {\bar{\hat{\mathbf{u}}}}_{p} } \right)} \right] = E\left[ {\left( {{\hat{\mathbf{u}}}_{p} - {\bar{\hat{\mathbf{u}}}}_{p} } \right)^{{\prime }} \left( {{\hat{\mathbf{u}}}_{p} - {\bar{\hat{\mathbf{u}}}}_{p} } \right)} \right]$, this has expectation:

$$\begin{aligned} acc_{p} & = E\left( {\rho_{T,p} } \right) \approx \frac{{\frac{1}{n}tr\left( {{\mathbf{SG}} - {\mathbf{SC}}_{p}^{uu} } \right)}}{{\sqrt {\frac{1}{n}tr\left( {{\mathbf{SG}}} \right)} \sqrt {\frac{1}{n}tr\left( {{\mathbf{SG}} - {\mathbf{SC}}_{p}^{uu} } \right)} }} \\ & = \frac{{\sqrt {\frac{1}{n}tr\left( {{\mathbf{SG}} - {\mathbf{SC}}_{p}^{uu} } \right)} }}{{\sqrt {\frac{1}{n}tr\left( {{\mathbf{SG}}} \right)} }} = \frac{{\sqrt {\left( {1 + \bar{F} - 2\bar{f}} \right)\sigma_{u,\infty }^{2} - \left( {\overline{{PEV_{p} }} - \overline{{PEC_{p} }} } \right)} }}{{\sqrt {\left( {1 + \bar{F} - 2\bar{f}} \right)\sigma_{u,\infty }^{2} } }} \\ \end{aligned}$$

The denominator $\left( {1 + \bar{F} - 2\bar{f}} \right)\sigma_{u,\infty }^{2}$ corresponds to the expected genetic variance in the focal population and takes the reduction in variance due to relationships $\left( {1 + \bar{F} - 2\bar{f}} \right)$ and selection ($\sigma_{u,\infty }^{2}$) into account. With all these elements, we can compute the expectation of the derived statistics, as done in the following.

### Derivation of statistics to test the quality of evaluation methods

#### Comparison of means of EBV from whole and EBV from partial data

$$\mu_{p,w} = \left( {\mathbf{1}^{{\prime }} \hat{\varvec{u}}_{p} - \mathbf{1}^{{\prime }} \hat{\varvec{u}}_{w} } \right)/n,$$

$$E\left( {\mu_{p,w} } \right) = E\left( {\frac{{\mathbf{1}^{{\prime }} \hat{\varvec{u}}_{p} }}{n}} \right) - E\left( {\frac{{\mathbf{1}^{{\prime }} \hat{\varvec{u}}_{w} }}{n}} \right) = \frac{1}{n}\left( {\mathbf{1}^{{\prime }} \mathbf{1}\mu_{p} - \mathbf{1}^{{\prime }} \mathbf{1}\mu_{w} } \right) = \mu_{p} - \mu_{w} .$$

#### Regression of EBV from whole data on EBV from partial data

The regression $b_{w,p} = \frac{{cov\left( {\hat{\varvec{u}}_{p} ,\hat{\varvec{u}}_{w} } \right)}}{{var\left( {\hat{\varvec{u}}_{p} } \right)}} = \frac{{\frac{1}{n}\left( {\hat{\varvec{u}}_{p} - \varvec{\bar{\hat{u}}}_{p} } \right)^{{\prime }} \left( {\hat{\varvec{u}}_{w} - \varvec{\bar{\hat{u}}}_{w} } \right)}}{{\frac{1}{n}\left( {\hat{\varvec{u}}_{p} - \varvec{\bar{\hat{u}}}_{p} } \right)^{{\prime }} \left( {\hat{\varvec{u}}_{p} - \varvec{\bar{\hat{u}}}_{p} } \right)}} = \frac{{\left( {\hat{\varvec{u}}_{p} - \varvec{\bar{\hat{u}}}_{p} } \right)^{{\prime }} \left( {\hat{\varvec{u}}_{w} - \varvec{\bar{\hat{u}}}_{w} } \right)}}{{\left( {\hat{\varvec{u}}_{p} - \varvec{\bar{\hat{u}}}_{p} } \right)^{{\prime }} \left( {\hat{\varvec{u}}_{p} - \varvec{\bar{\hat{u}}}_{p} } \right)}}$ is composed of two quadratic forms. When assuming that the expectation of the ratio is equal to the ratio of the expectations,

$$E\left( {b_{w,p} } \right) \approx \frac{{\left( {1 + \bar{F} - 2\bar{f}} \right)\sigma_{u,\infty }^{2} - \left( {\overline{{PEV_{p} }} - \overline{{PEC_{p} }} } \right)}}{{\left( {1 + \bar{F} - 2\bar{f}} \right)\sigma_{u,\infty }^{2} - \left( {\overline{{PEV_{p} }} - \overline{{PEC_{p} }} } \right)}} = 1.$$

Note that this expectation involves PEV and off-diagonal PEC. Importantly, it must hold that $Var\left( {{\hat{\mathbf{u}}}_{p} } \right) = Cov\left( {{\hat{\mathbf{u}}}_{p} ,{\hat{\mathbf{u}}}_{w}^{{\prime }} } \right)$ (as usually assumed).

#### Correlation of EBV from whole and EBV from partial data

$$\rho_{w,p} = \frac{{cov\left( {{\hat{\mathbf{u}}}_{p} ,{\hat{\mathbf{u}}}_{w} } \right)}}{{\sqrt {var\left( {{\hat{\mathbf{u}}}_{w} } \right)var\left( {{\hat{\mathbf{u}}}_{p} } \right)} }} = \frac{{ \frac{1}{n}\left( {{\hat{\mathbf{u}}}_{p} - {\bar{\hat{\mathbf{u}}}}_{p} } \right)^{{\prime }} \left( {{\hat{\mathbf{u}}}_{w} - {\bar{\hat{\mathbf{u}}}}_{w} } \right)}}{{\sqrt { \frac{1}{n}\left( {\hat{\varvec{u}}_{w} - \varvec{\bar{\hat{u}}}_{w} } \right)^{ '} \left( {\hat{\varvec{u}}_{w} - \varvec{\bar{\hat{u}}}_{w} } \right) \frac{1}{n}\left( {\hat{\varvec{u}}_{p} - \varvec{\bar{\hat{u}}}_{p} } \right)^{{\prime }} \left( {\hat{\varvec{u}}_{p} - \varvec{\bar{\hat{u}}}_{p} } \right)} }}.$$

This statistic is composed of three quadratic forms and assuming that the square root of the expectation is equal to the expectation of the root, it gives:

$$\begin{aligned} E\left( {\rho_{w,p} } \right) & \approx \frac{{\frac{1}{n}tr\left( {{\mathbf{SG}} - {\mathbf{SC}}_{p}^{uu} } \right)}}{{\sqrt {\frac{1}{n}tr\left( {{\mathbf{SG}} - {\mathbf{SC}}_{w}^{uu} } \right)} \sqrt {\frac{1}{n}tr\left( {{\mathbf{SG}} - {\mathbf{S}}\varvec{C}_{p}^{uu} } \right)} }} = \frac{{\sqrt {\frac{1}{n}tr\left( {{\mathbf{SG}} - {\mathbf{SC}}_{p}^{uu} } \right)} }}{{\sqrt {\frac{1}{n}tr\left( {{\mathbf{SG}} - {\mathbf{SC}}_{w}^{uu} } \right)} }} \\ & = \sqrt {\frac{{\left( {1 + \bar{F} - 2\bar{f}} \right)\sigma_{u,\infty }^{2} - \left( {\overline{{PEV_{p} }} - \overline{{PEC_{p} }} } \right)}}{{\left( {1 + \bar{F} - 2\bar{f}} \right)\sigma_{u,\infty }^{2} - \left( {\overline{{PEV_{w} }} - \overline{{PEC_{w} }} } \right)}}} = \frac{{\sqrt {1 - \frac{{\left( {\overline{{PEV_{p} }} - \overline{{PEC_{p} }} } \right)}}{{\left( {1 + \bar{F} - 2\bar{f}} \right)\sigma_{u,\infty }^{2} }}} }}{{\sqrt {1 - \frac{{\left( {\overline{{PEV_{w} }} - \overline{{PEC_{w} }} } \right)}}{{\left( {1 + \bar{F} - 2\bar{f}} \right)\sigma_{u,\infty }^{2} }}} }} \\ & = \frac{{acc_{p} }}{{acc_{w} }}. \\ \end{aligned}$$

Therefore, $\rho_{w,p}$ is a direct estimator of the increase in population accuracy of EBV from partial to whole data, $\frac{{acc_{p} }}{{acc_{w} }}$.

#### Estimation of accuracy from the covariance of EBV based on whole and EBV based on partial data

We can get a direct estimator of accuracy (and not of ratios of accuracies) from $cov\left( {{\hat{\mathbf{u}}}_{p} ,{\hat{\mathbf{u}}}_{w} } \right) = \frac{1}{n}\left( {{\hat{\mathbf{u}}}_{p} - {\bar{\hat{\mathbf{u}}}}_{p} } \right)^{'} \left( {{\hat{\mathbf{u}}}_{w} - {\bar{\hat{\mathbf{u}}}}_{w} } \right)$, from which we can derive the statistic:

$$\rho_{{cov_{w,p} }}^{2} = \frac{{cov\left( {{\hat{\mathbf{u}}}_{p} ,{\hat{\mathbf{u}}}_{w} } \right)}}{{\left( {1 + \bar{F} - 2\bar{f}} \right)\sigma_{u,\infty }^{2} }}$$

with expectation $acc_{p}^{2}$ as follows

$$acc_{p}^{2} = E\left( {\rho_{{cov_{w,p} }}^{2} } \right) = \frac{{E\left( {\frac{1}{n}\left( {{\hat{\mathbf{u}}}_{p} - {\bar{\hat{\mathbf{u}}}}_{p} } \right)^{{\prime }} \left( {{\hat{\mathbf{u}}}_{w} - {\bar{\hat{\mathbf{u}}}}_{w} } \right)} \right)}}{{\left( {1 + \bar{F} - 2\bar{f}} \right)\sigma_{u,\infty }^{2} }} \approx \frac{{1 - \overline{{PEV_{p} }} + \overline{{PEC_{p} }} }}{{\left( {1 + \bar{F} - 2\bar{f}} \right)\sigma_{u,\infty }^{2} }}.$$

Thus, $\rho_{{cov_{w,p} }}^{2}$ is a direct estimate of squared population accuracy of EBV based on partial data, which we call $\rho_{{cov_{w,p} }}^{2}$ since it is an estimator of the squared accuracy (a squared correlation) based on the covariance between ${\hat{\mathbf{u}}}_{p}$ and ${\hat{\mathbf{u}}}_{w}$. This statistic requires an estimator of $\sigma_{u,\infty }^{2}$ that can be obtained by modelling the selection scheme [18] or be explicitly estimated [34].

#### Regression of EBV from partial data on EBV from whole data

$$b_{p,w} = \frac{{\frac{1}{n}\left( {{\hat{\mathbf{u}}}_{p} - {\bar{\hat{\mathbf{u}}}}_{p} } \right)^{{\prime }} \left( {{\hat{\mathbf{u}}}_{w} - {\bar{\hat{\mathbf{u}}}}_{w} } \right)}}{{\frac{1}{n}\left( {{\hat{\mathbf{u}}}_{w} - {\bar{\hat{\mathbf{u}}}}_{w} } \right)^{{\prime }} \left( {{\hat{\mathbf{u}}}_{w} - {\bar{\hat{\mathbf{u}}}}_{w} } \right)}} = \frac{{cov\left( {{\hat{\mathbf{u}}}_{p} ,{\hat{\mathbf{u}}}_{w} } \right)}}{{var\left( {{\hat{\mathbf{u}}}_{w} } \right)}},$$

with expectation:

$$E\left( {b_{p,w} } \right) \approx \frac{{\frac{1}{n}tr\left( {{\mathbf{CG}} - {\mathbf{CC}}_{p}^{uu} } \right)}}{{\frac{1}{n}tr\left( {{\mathbf{CG}} - {\mathbf{CC}}_{w}^{uu} } \right)}} = \frac{{\left( {1 + \bar{F} - 2\bar{f}} \right)\sigma_{u,\infty }^{2} - \left( {\overline{{PEV_{p} }} - \overline{{PEC_{p} }} } \right)}}{{\left( {1 + \bar{F} - 2\bar{f}} \right)\sigma_{u,\infty }^{2} - \left( {\overline{{PEV_{w} }} - \overline{{PEC_{w} }} } \right)}},$$

which is a function of squared population accuracies, i.e. $E\left( {b_{p,w} } \right) = \frac{{acc_{p}^{2} }}{{acc_{w}^{2} }}$. In addition, $E\left( {\rho_{w,p} } \right) = \sqrt {E\left( {b_{p,w} } \right)}$, although $\rho_{w,p}$ and $\sqrt {b_{p,w} }$ need not be equal for single realizations, i.e. for the analysis of one particular dataset.

#### Effect of over/underdispersion of breeding values on statistics

Statistics used to compute slopes and accuracies deal well with regular bias (${\bar{\hat{\mathbf{u}}}}_{p} \ne {\bar{\hat{\mathbf{u}}}}_{w}$) because the ${\hat{\mathbf{u}}}_{p}$ and ${\hat{\mathbf{u}}}_{w}$ are centered. However, overdispersion (inflation) of EBV is a frequent phenomenon [6]. To consider a simple case, assume that EBV based on partial and whole data are uniformly scaled by regression coefficients $\theta_{p}^{2}$ and $\theta_{w}^{2}$, with $\theta_{p}^{2} > \theta_{w}^{2} \ge 1$ (i.e., there is more overdispersion with less data or with old data), resulting in:

$$var\left( {\begin{array}{*{20}c} {\hat{\varvec{u}}_{p} } \\ {\hat{\varvec{u}}_{w} } \\ \varvec{u} \\ \end{array} } \right) = \left( {\begin{array}{*{20}c} {\theta_{p}^{2} \left( {{\mathbf{G}} - {\mathbf{C}}_{p}^{uu} } \right)} & {\theta_{p} \theta_{w} \left( {{\mathbf{G}} - {\mathbf{C}}_{p}^{uu} } \right)} & {\theta_{p} \left( {{\mathbf{G}} - {\mathbf{C}}_{p}^{uu} } \right)} \\ {\theta_{p} \theta_{w} \left( {{\mathbf{G}} - {\mathbf{C}}_{p}^{uu} } \right)} & {\theta_{w}^{2} \left( {{\mathbf{G}} - {\mathbf{C}}_{w}^{uu} } \right)} & {\theta_{w} \left( {{\mathbf{G}} - {\mathbf{C}}_{w}^{uu} } \right)} \\ {\theta_{p} \left( {{\mathbf{G}} - {\mathbf{C}}_{p}^{uu} } \right)} & {\theta_{w} \left( {{\mathbf{G}} - {\mathbf{C}}_{w}^{uu} } \right)} & {\mathbf{G}} \\ \end{array} } \right),$$

yielding, e.g.,

$$\begin{aligned} E\left( {cov\left( {\hat{u}_{p} ,\hat{u}_{w} } \right)} \right) & = E\left( {\frac{1}{n}\left( {{\hat{\mathbf{u}}}_{p} - {\bar{\hat{\mathbf{u}}}}_{p} } \right)^{'} \left( {{\hat{\mathbf{u}}}_{w} - {\bar{\hat{\mathbf{u}}}}_{w} } \right)} \right) \approx \frac{{\theta_{p} \theta_{w} }}{n}tr\left( {{\mathbf{SG}} - {\mathbf{SC}}_{p}^{uu} } \right) \\ & = \theta_{p} \theta_{w} \left\{ {\overline{{diag\left( {\mathbf{G}} \right)}} - {\bar{\mathbf{G}}} - \left( {\overline{{diag\left( {{\mathbf{C}}_{p}^{uu} } \right)}} - \overline{{\left( {{\mathbf{C}}_{p}^{uu} } \right)}} } \right)} \right\} \\ & = \theta_{p} \theta_{w} \left\{ {\left( {1 + \bar{F} - 2\bar{f}} \right)\sigma_{u,\infty }^{2} - \left( {\overline{{PEV_{p} }} - \overline{{PEC_{p} }} } \right)} \right\}. \\ \end{aligned}$$

The regression of EBV from whole on partial data, $b_{w,p} = \frac{{\left( {{\hat{\mathbf{u}}}_{p} - {\bar{\hat{\mathbf{u}}}}_{p} } \right)^{{\prime }} \left( {{\hat{\mathbf{u}}}_{w} - {\bar{\hat{\mathbf{u}}}}_{w} } \right)}}{{\left( {{\hat{\mathbf{u}}}_{p} - {\bar{\hat{\mathbf{u}}}}_{p} } \right)^{{\prime }} \left( {{\hat{\mathbf{u}}}_{p} - {\bar{\hat{\mathbf{u}}}}_{p} } \right)}}$, yields on expectation $E\left( {b_{w,p} } \right) \approx \frac{{\theta_{w} }}{{\theta_{p} }}\frac{{\left( {1 + \bar{F} - 2\bar{f}} \right)\sigma_{u,\infty }^{2} - \left( {\overline{{PEV_{p} }} - \overline{{COPEV_{p} }} } \right)}}{{\left( {1 + \bar{F} - 2\bar{f}} \right)\sigma_{u,\infty }^{2} - \left( {\overline{{PEV_{p} }} - \overline{{COPEV_{p} }} } \right)}} = \frac{{\theta_{w} }}{{\theta_{p} }}$, which is not equal to 1 but equal to the ratio of dispersions. Thus, a value of $b_{w,p} < 1$ (as often observed for genomic predictions) may indicate overdispersion of EBV based on partial data but also underdispersion of EBV based on whole data.

The reverse regression of EBV from partial on whole data, $b_{p,w} = \frac{{\frac{1}{n}\left( {{\hat{\mathbf{u}}}_{p} - {\bar{\hat{\mathbf{u}}}}_{p} } \right)^{{\prime }} \left( {{\hat{\mathbf{u}}}_{w} - {\bar{\hat{\mathbf{u}}}}_{w} } \right)}}{{\frac{1}{n}\left( {{\hat{\mathbf{u}}}_{w} - {\bar{\hat{\mathbf{u}}}}_{w} } \right)^{{\prime }} \left( {{\hat{\mathbf{u}}}_{w} - {\bar{\hat{\mathbf{u}}}}_{w} } \right)}},$ yields on expectation $E\left( {b_{p,w} } \right) \approx \frac{{\theta_{p} }}{{\theta_{w} }}\frac{{\left( {1 + \bar{F} - 2\bar{f}} \right)\sigma_{u}^{2} - \left( {\overline{{PEV_{p} }} - \overline{{PEC_{p} }} } \right)}}{{\left( {1 + \bar{F} - 2\bar{f}} \right)\sigma_{u}^{2} - \left( {\overline{{PEV_{w} }} - \overline{{PEC_{w} }} } \right)}} = \frac{{\theta_{p} }}{{\theta_{w} }}\frac{{acc_{p}^{2} }}{{acc_{w}^{2} }}$, which is a ratio of dispersions and reliabilities.

Finally, the correlation $\rho_{w,p} = \frac{{ \left( {{\hat{\mathbf{u}}}_{p} - {\bar{\hat{\mathbf{u}}}}_{p} } \right)^{{\prime }} \left( {{\hat{\mathbf{u}}}_{w} - {\bar{\hat{\mathbf{u}}}}_{w} } \right)}}{{\sqrt { \left( {{\hat{\mathbf{u}}}_{w} - {\bar{\hat{\mathbf{u}}}}_{w} } \right)^{ '} \left( {{\hat{\mathbf{u}}}_{w} - {\bar{\hat{\mathbf{u}}}}_{w} } \right) \left( {{\hat{\mathbf{u}}}_{p} - {\bar{\hat{\mathbf{u}}}}_{p} } \right)^{{\prime }} \left( {{\hat{\mathbf{u}}}_{p} - {\bar{\hat{\mathbf{u}}}}_{p} } \right)} }}$ has the following expected value:

$$\begin{aligned} E\left( {\rho_{w,p} } \right) & \approx \frac{{\theta_{p} \theta_{w} \frac{1}{n}tr\left( {{\mathbf{SG}} - {\mathbf{SC}}_{p}^{uu} } \right)}}{{\sqrt {\theta_{w}^{2} \frac{1}{n}tr\left( {{\mathbf{SG}} - {\mathbf{SC}}_{w}^{uu} } \right)} \sqrt {\theta_{p}^{2} \frac{1}{n}tr\left( {{\mathbf{SG}} - {\mathbf{SC}}_{p}^{uu} } \right)} }} \\ & = \frac{{\sqrt {\frac{1}{n}tr\left( {{\mathbf{SG}} - {\mathbf{SC}}_{p}^{uu} } \right)} }}{{\sqrt {\frac{1}{n}tr\left( {{\mathbf{SG}} - {\mathbf{SC}}_{w}^{uu} } \right)} }} = \sqrt {\frac{{\left( {1 + \bar{F} - 2\bar{f}} \right)\sigma_{u,\infty }^{2} - \left( {\overline{{PEV_{p} }} - \overline{{PEC_{p} }} } \right)}}{{\left( {1 + \bar{F} - 2\bar{f}} \right)\sigma_{u,\infty }^{2} - \left( {\overline{{PEV_{w} }} - \overline{{PEC_{w} }} } \right)}}} = \frac{{acc_{p} }}{{acc_{w} }}, \\ \end{aligned}$$

retrieving a ratio of accuracies. Thus, the statistic $\rho_{w,p}$ (correlation of “whole data” and “partial data” EBV) is an estimator of change in accuracy and is not affected by this very simplistic form of overdispersion.

Note that equivalent biases result when $\theta_{w}^{2} > \theta_{p}^{2} \ge 1$, i.e. when there is more overdispersion with more data or with recent data. Thus, regression of EBV from whole on partial data informs about over/underdispersion, regression of EBV from partial on whole data can be interpreted as a function of accuracies, and the correlation of EBV from partial and whole data is useful as a ratio of accuracies.

### Predictivity: correlation of EBV with precorrected data

A very common strategy in cross-validation tests is to compare predictions of EBV with precorrected phenotypes for the predicted individuals [9, 10], i.e. using $r\left( {\varvec{y}_{new}^{*} ,\hat{\varvec{u}}_{p} } \right)$, where $\varvec{y}_{new}^{*}$ is the precorrected “new” data available in the whole data. It is, however, not clear whether this is a valid estimator of accuracy and what the effect of precorrection is. Here we derive some results that show that the use of precorrected data can be problematic in some cases: many levels of the main environmental effect or wrong variance components.

Precorrected data are obtained with the whole dataset using ${\mathbf{y}}^{ *} = {\mathbf{y}} - {{\mathbf{X}}\hat{\boldsymbol{\beta} }} = \left( {{\mathbf{I}} - {\mathbf{X}}\left( {{\mathbf{X}}^{{\prime }} {\mathbf{V}}^{ - 1} {\mathbf{X}}} \right)^{ - } {\mathbf{X}}^{{\prime }} {\mathbf{V}}^{ - 1} } \right){\mathbf{y}}$, where ${\hat{\boldsymbol{\beta }}}$ is typically a BLUE estimator of fixed effects. In fact, $Var\left( {{\mathbf{y}}^{*} } \right) = {\mathbf{VPV}}$ for ${\mathbf{P}} = {\mathbf{V}}^{ - 1} \left( {{\mathbf{I}} - {\mathbf{X}}\left( {{\mathbf{X}}^{{\prime }} {\mathbf{V}}^{ - 1} {\mathbf{X}}} \right)^{ - } {\mathbf{X}}^{{\prime }} {\mathbf{V}}^{ - 1} } \right)$ [36], which leads to:

$$\begin{aligned} Var\left( {{\mathbf{y}}^{*} } \right) & = {\mathbf{VPV}} = {\mathbf{VV}}^{ - 1} \left( {{\mathbf{I}} - {\mathbf{X}}\left( {{\mathbf{X^{\prime}V}}^{ - 1} {\mathbf{X}}} \right)^{ - } {\mathbf{X^{\prime}V}}^{ - 1} } \right){\mathbf{V}} \\ & = {\mathbf{V}} - {\mathbf{X}}\left( {{\mathbf{X}}^{{\prime }} {\mathbf{V}}^{ - 1} {\mathbf{X}}} \right)^{ - } {\mathbf{X}}^{{\prime }} = {\mathbf{V}} - {\mathbf{XC}}_{w}^{{\varvec{\beta \beta }}} {\mathbf{X}}^{{\prime }} = {\mathbf{R}} + {\mathbf{ZGZ}}^{{\prime }} - {\mathbf{XC}}_{w}^{{\varvec{\beta \beta }}} \varvec{X}^{{\prime }} , \\ \end{aligned}$$

where ${\mathbf{C}}_{w}^{{\varvec{\beta \beta }}}$ is the PEV of fixed effects in ${\varvec{\upbeta}}$ obtained from analysis of the whole dataset.

Now, we will consider only new data that are not in the partial dataset and assume for simplicity one record per individual. We further assume that the new data are only affected by a single fixed effect (say contemporary group), such that:

$$Var\left( {{\mathbf{y}}_{new}^{*} } \right) = {\mathbf{R}} + {\mathbf{G}} - {\mathbf{XC}}_{w}^{{\varvec{\beta \beta }}} {\mathbf{X}}^{{\prime }} .$$

The covariance of EBV with ${\mathbf{y}}_{new}^{*}$ can be obtained as follows:

$$\begin{aligned} Cov\left( {{\hat{\mathbf{u}}}_{p} ,{\mathbf{y}}_{new}^{*} } \right) & = Cov\left( {\hat{\mathbf{u}}}_{p} ,{\mathbf{y}}_{new} - {\mathbf{X}}{\hat{\boldsymbol{\upbeta }}}\right) = Cov\left( {{\hat{\mathbf{u}}}_{p} ,{\mathbf{y}}_{new} } \right) - Cov\left( {{\hat{\mathbf{u}}}_{p} ,{{\mathbf{X}}{\hat{\boldsymbol{\upbeta}} }}} \right) \\ & = Cov\left( {{\hat{\mathbf{u}}}_{p} ,{{\mathbf{y}}}_{new} } \right) = Cov\left( {{\hat{\mathbf{u}}}_{p} ,{\mathbf{u}} + {\mathbf{e}}_{new} } \right) = Cov\left( {{\hat{\mathbf{u}}}_{p} ,{\mathbf{u}}} \right) + Cov\left( {{\hat{\mathbf{u}}}_{p} ,{\mathbf{e}}_{new} } \right). \\ \end{aligned}$$

Because by orthogonality, $Cov\left( {\hat{\mathbf{u}}}_{p} ,{\mathbf{X}}{\hat{\boldsymbol{\beta }}} \right) = 0$ ([37] equation 5.28), and where $Cov\left( {{\hat{\mathbf{u}}}_{p} ,{\mathbf{u}}} \right) = {\mathbf{G}} - {\mathbf{C}}_{p}^{uu}$, and $Cov\left( {{\hat{\mathbf{u}}}_{p} ,{\mathbf{e}}_{new} } \right) = 0$, the latter because EBV based on partial data do not influence ${\mathbf{e}}_{new}$ (again, assuming there is no effect of selection).

Therefore, $$var\left( {\begin{array}{*{20}c} {{\hat{\mathbf{u}}}_{p} } \\ {{\mathbf{y}}_{new}^{*} } \\ \end{array} } \right) = \left( {\begin{array}{*{20}c} {{\mathbf{G}} - {\mathbf{C}}_{p}^{uu} } & {{\mathbf{G}} - {\mathbf{C}}_{p}^{uu} } \\ {{\mathbf{G}} - {\mathbf{C}}_{p}^{uu} } & {{\mathbf{R}} + {\mathbf{G}} - {\mathbf{XC}}_{w}^{{\varvec{\beta \beta }}} {\mathbf{X}}^{{\prime }} } \\ \end{array} } \right)$$,

which yields the following expectations for $n$ individuals:

$$E\left( {b_{{y_{new}^{*} ,\hat{u}_{p} }} } \right) = \frac{{\frac{1}{n}tr\left( {{\mathbf{SG}} - {\mathbf{SC}}_{p}^{uu} } \right)}}{{\frac{1}{n}tr\left( {{\mathbf{SG}} - {\mathbf{SC}}_{p}^{uu} } \right)}} = 1,$$

which is equal to 1 as expected, and the correlation is equal to:

$$\rho_{{y_{new}^{*} ,\hat{u}_{p} }} = \frac{{ \left( {{\hat{\mathbf{u}}}_{p} - {\bar{\hat{\mathbf{u}}}}_{p} } \right)^{ '} \left( {{\mathbf{y}}_{new}^{*} - {\bar{\mathbf{y}}}_{new}^{*} } \right)}}{{\sqrt { \left( {{\mathbf{y}}_{new}^{*} - {\bar{\mathbf{y}}}_{new}^{*} } \right)^{'} \left( {{\mathbf{y}}_{new}^{*} - {\bar{\mathbf{y}}}_{new}^{*} } \right)\left( {{\hat{\mathbf{u}}}_{p} - {\bar{\hat{\mathbf{u}}}}_{p} } \right)^{ '} \left( {{\hat{\mathbf{u}}}_{p} - {\bar{\hat{\mathbf{u}}}}_{p} } \right)} }},$$

with expectation:

$$\begin{aligned} E\left( {\rho_{{y_{new}^{*} ,\hat{u}_{p} }} } \right) & = \frac{{\frac{1}{n}tr\left( {{\mathbf{SG}} - {\mathbf{SC}}_{p}^{uu} } \right)}}{{\sqrt {\frac{1}{n}tr\left( {{\mathbf{S}}\left( {{\mathbf{R}} + {\mathbf{G}} - {\mathbf{XC}}_{w}^{{\varvec{\beta \beta }}} {\mathbf{X^{\prime}}}} \right)} \right)} \sqrt {\frac{1}{n}tr\left( {{\mathbf{SG}} - {\mathbf{SC}}_{p}^{uu} } \right)} }} \\ & = \frac{{\sqrt {\frac{1}{n}tr\left( {{\mathbf{SG}} - {\mathbf{SC}}_{p}^{uu} } \right)} }}{{\sqrt {\frac{1}{n}tr\left( {{\mathbf{S}}\left( {{\mathbf{R}} + {\mathbf{G}} - {\mathbf{XC}}_{w}^{{\varvec{\beta \beta }}} {\mathbf{X}}^{{\prime }} } \right)} \right)} }} = \frac{{\sqrt {\left( {1 + \bar{F} - 2\bar{f}} \right)\sigma_{u,\infty }^{2} - \left( {\overline{{PEV_{p} }} - \overline{{PEC_{p} }} } \right)} }}{{\sqrt {\sigma_{e}^{2} + \left( {1 + \bar{F} - 2\bar{f}} \right)\sigma_{u,\infty }^{2} - \frac{1}{n}tr\left( {{\mathbf{SXS}}_{w}^{{\varvec{\beta \beta }}} {\mathbf{X}}^{{\prime }} } \right)} }}. \\ \end{aligned}$$

Thus, the cross-validation correlation of EBV with precorrected phenotypes depends on population accuracy, heritability, and errors in estimates of fixed effects. If fixed effects are estimated with high precision ${\mathbf{C}}_{w}^{{\varvec{\beta \beta }}} \approx 0$ and off-diagonals (both in relationships and in PEV) are negligible, then:

$$E\left( {\rho_{{y_{new}^{*} ,\hat{u}_{p} }} } \right) = \frac{{\sqrt {\sigma_{u,\infty }^{2} - PEV} }}{{\sqrt {\sigma_{e}^{2} + \sigma_{u,\infty }^{2} } }} = \sqrt {\frac{{\sigma_{u,\infty }^{2} - PEV}}{{\sigma_{u,\infty }^{2} + \sigma_{e}^{2} }}} .$$

If we divide the square of this by the population heritability $h_{\infty }^{2} = \frac{{\sigma_{u,\infty }^{2} }}{{\sigma_{u,\infty }^{2} + \sigma_{e}^{2} }}$ (i.e. in the selected population, not in the base population):

$$\frac{{\frac{{\sigma_{u,\infty }^{2} - PEV}}{{\sigma_{u,\infty }^{2} + \sigma_{e}^{2} }}}}{{\frac{{\sigma_{u,\infty }^{2} }}{{\sigma_{u,\infty }^{2} + \sigma_{e}^{2} }}}} = \frac{{\sigma_{u,\infty }^{2} - PEV}}{{\sigma_{u,\infty }^{2} }} = acc_{p}^{2} ,$$

and therefore, $$E\left( {\rho_{{y_{new}^{*} ,\hat{u}_{p} }} } \right) = \frac{acc}{{h_{\infty } }}.$$

Thus, if there has been no selection, we can estimate accuracy from cross-validation as: $\widehat{acc} \approx \frac{{\rho_{{y_{new}^{*} ,\hat{u}_{p} }} }}{h}$ where $h^{2}$ is heritability in the base population [9]. However, if there has been selection, using $\widehat{acc} \approx \frac{{\rho_{{y_{new}^{*} ,\hat{u}_{p} }} }}{h}$ underestimates population accuracy because $\sigma_{u,\infty }^{2} < \sigma_{u}^{2}$ and $h_{\infty }^{2} < h^{2}$. Using the “dairy” example in [18], $\sigma_{u}^{2} = h^{2} = 0.3$ and $\sigma_{u,\infty }^{2} = 0.18$, such that $h_{\infty }^{2} = 0.20$. If the observed $\rho_{{y_{new}^{*} ,\hat{u}_{p} }} = 0.3$, this yields (biased) $\widehat{acc} \approx \frac{{\rho_{{y_{new}^{*} ,\hat{u}_{p} }} }}{h} = 0.55$ and (correct) $\widehat{acc} \approx \frac{{\rho_{{y_{new}^{*} ,\hat{u}_{p} }} }}{{h_{\infty } }} = 0.67$. The latter can, in turn, be translated to an “unselected accuracy” of 0.82 [18, 27].

There is a second and not negligible source of bias due to ${\mathbf{C}}_{w}^{{\varvec{\beta \beta }}} \ne 0$. For a single fixed effect, matrix ${\mathbf{XC}}_{w}^{{\varvec{\beta \beta }}} {\mathbf{X}}^{{\prime }}$ contains $var\left( {\hat{\beta }_{i} } \right)$ (the variance of the estimate of the effect that affects the $i$-th record) on the diagonal and $cov\left( {\hat{\beta }_{i} ,\hat{\beta }_{j} } \right)$ on off-diagonals (the covariance of the estimates of the effects that affect the $i$-th and $j$-th records). We will assume that covariances of estimates across levels of the fixed effect are negligible (this is not true if relatives are spread across fixed effects). For a balanced design with $n$ records in ${\mathbf{y}}_{new}^{*}$, $n_{i} = n/m$ records for each of the $m$ levels of the fixed effect, and with records ordered within level, the structure of ${\mathbf{XC}}_{w}^{{\varvec{\beta \beta }}} {\mathbf{X}}^{{\prime }}$ is:

$$\left( {\begin{array}{*{20}c} {\left( {\begin{array}{*{20}c} {var\left( {\hat{\beta }_{1} } \right)} & {var\left( {\hat{\beta }_{1} } \right)} & \ldots \\ {var\left( {\hat{\beta }_{1} } \right)} & {var\left( {\hat{\beta }_{1} } \right)} & \ldots \\ \ldots & \ldots & \ldots \\ \end{array} } \right)} & { \approx {\mathbf{0}}} & \ldots & { \approx {\mathbf{0}}} \\ { \approx {\mathbf{0}}} & {\left( {\begin{array}{*{20}c} {var\left( {\hat{\beta }_{2} } \right)} & {var\left( {\hat{\beta }_{2} } \right)} & \ldots \\ {var\left( {\hat{\beta }_{2} } \right)} & {var\left( {\hat{\beta }_{2} } \right)} & \ldots \\ \ldots & \ldots & \ldots \\ \end{array} } \right)} & \ldots & \varvec{ } \\ \ldots & \ldots & \ldots & \\ { \approx {\mathbf{0}}} & & & {\left( {\begin{array}{*{20}c} {var\left( {\hat{\beta }_{m} } \right)} & {var\left( {\hat{\beta }_{m} } \right)} & \ldots \\ {var\left( {\hat{\beta }_{m} } \right)} & {var\left( {\hat{\beta }_{m} } \right)} & \ldots \\ \ldots & \ldots & \ldots \\ \end{array} } \right)} \\ \end{array} } \right)$$

where $var\left( {\hat{\beta }_{1} } \right) = var\left( {\hat{\beta }_{2} } \right) = \ldots = var\left( {\hat{\beta }_{m} } \right) = var\left( {\hat{\beta }} \right)$. Also, we will assume that $var\left( {\hat{\beta }_{i} } \right) = \frac{{\sigma_{u,\infty }^{2} + \sigma_{e}^{2} }}{{n_{i} }}$, in other words, relationships add little information to estimates of the fixed effect. This results in $\overline{{diag\left( {{\mathbf{XC}}_{w}^{{\varvec{\beta \beta }}} {\mathbf{X}}^{{\prime }} } \right)}} = var\left( {\hat{\beta }} \right) = \frac{{\sigma_{u,\infty }^{2} + \sigma_{e}^{2} }}{{n_{i} }}$ and $\overline{{\left( {{\mathbf{XC}}_{w}^{{\varvec{\beta \beta }}} {\mathbf{X}}^{{\prime }} } \right)}} = \frac{{\sigma_{u,\infty }^{2} + \sigma_{e}^{2} }}{n}$, which results in $\frac{1}{n}tr\left( {{\mathbf{SXC}}_{w}^{{\varvec{\beta \beta }}} {\mathbf{X}}^{{\prime }} } \right) = \frac{{\sigma_{u,\infty }^{2} + \sigma_{e}^{2} }}{{n_{i} }}\left( {1 - \frac{1}{m}} \right)$. Plugging this expression in $E\left( {\rho_{{y_{new}^{*} ,\hat{u}_{p} }} } \right)$ above and ignoring off-diagonals results in:

$$\frac{{E\left( {\rho_{{y_{new}^{*} ,\hat{u}_{p} }} } \right)}}{h} \approx acc_{p} \left( {1 + \frac{m - 1}{{n - \left( {m - 1} \right)}}} \right).$$

This results in overestimation of the accuracy of $\frac{m - 1}{{n - \left( {m - 1} \right)}}$, which does not disappear with high values of *n*. Thus, if there are several levels of the fixed effect, the estimate of the cross-validation accuracy will have an upward bias, which is greater for a smaller number of records per contemporary group. This bias is due to the assumption that the precorrection is perfect. For instance, for $n = 500$ and 25 contemporary groups, the bias is an extra 5% apparent accuracy. For $n$ “large” and $m$ “not small”, bias is approximately $\frac{1}{{n_{i} }}$, i.e., inversely proportional to the size of the contemporary group, which does not disappear with increasing $n$.

### Comparison with current Interbull validation procedures

The Interbull method [6] uses a simple regression that can be written as $2{\mathbf{DYD}} = 1b_{0} + b_{1} {\hat{\mathbf{u}}}_{p} + \varvec{\epsilon}$, where ${\text{DYD}}$ are daughter yield deviations (computed with the whole dataset) and act as pseudo-data for bulls. Elements of $\varvec{\epsilon}$ are assumed to be independent across bulls with variance inversely proportional to the equivalent number of daughters (this can be viewed as DYD having different heritabilities across bulls). Thus, this setting is similar to the previous section on predictivity. The above proofs apply and the expected value of $b_{1}$ is 1, although, using $\hat{a} = \overline{\text{DYD}} - \hat{b}_{1} {\bar{\hat{\mathbf{u}}}}_{p}$ does *not* yield a correct estimate of $\mu_{p} - \mu_{w}$, i.e. bias, unless $b_{1} = 1$. Also, the expected value of $r^{2} \left( {\hat{u}_{p} ,{\text{DYD}}} \right)$ is $\frac{{acc^{2} }}{{\overline{rel} }}$, where $\overline{rel}$ is the average reliability of the EBV of bulls based on progeny. Here, as in the analysis on predictivity, off-diagonals are ignored, which should not affect results if progeny numbers are large enough.

### Markers considered as “new” data: pedigree BLUP and (SS)GBLUP

The addition of marker genotypes to a pedigree-based BLUP genetic evaluation can also be viewed as having “more data”, e.g. on a correlated trait [38, 39]. Thus, a way to check the increase in accuracy from adding marker information (e.g. from BLUP to GBLUP) is to view the data with marker genotypes as “whole” and the data without markers as “partial”. Using $G$ to refer to EBV with markers and $A$ to EBV without markers, this yields:

$$\rho_{A,G} = \frac{{ \left( {{\hat{\mathbf{u}}}_{A} - {\bar{\hat{\mathbf{u}}}}_{A} } \right)^{{\prime }} \left( {{\hat{\mathbf{u}}}_{G} - {\bar{\hat{\mathbf{u}}}}_{G} } \right)}}{{\sqrt { \left( {{\hat{\mathbf{u}}}_{G} - {\bar{\hat{\mathbf{u}}}}_{G} } \right)^{{\prime }} \left( {{\hat{\mathbf{u}}}_{G} - {\bar{\hat{\mathbf{u}}}}_{G} } \right) \left( {{\hat{\mathbf{u}}}_{A} - {\bar{\hat{\mathbf{u}}}}_{A} } \right)^{{\prime }} \left( {{\hat{\mathbf{u}}}_{A} - {\bar{\hat{\mathbf{u}}}}_{A} } \right)} }} = \frac{{acc_{A} }}{{acc_{G} }},$$

i.e., the lower the correlation between genomic EBV and pedigree EBV, the higher the extra accuracy from genomic data. This assumes that $Cov\left( {{\hat{\mathbf{u}}}_{G} ,{\hat{\mathbf{u}}}_{A} } \right) = Var\left( {{\hat{\mathbf{u}}}_{A} } \right)$, as assumed by [39], which sounds reasonable but has been formally proved only for a single marker that is fitted as a correlated trait [38].

The procedure above uses the same phenotypes for the evaluations with either $G$ or $A$. An alternative procedure may be to compare the increase in accuracy from “partial” to “whole” in both approaches. In this case, to compare EBV from a genomic-based method (GBLUP or SSGBLUP) with EBV from a pedigree-based method (PBLUP), we suggest the following procedure:

1. Compute EBV with all data (“whole”) using the method that is deemed to be optimal; we will assume that this is GBLUP.
2. Choose a cutoff date and create a partial dataset by setting phenotypes after cutoff date to missing;
3. Compute GEBV based on the partial data using GBLUP;
4. For “focal” individuals (i.e., the validation cohort): compute statistics $\mu_{p,w}^{GBLUP}$, $b_{w,p}^{GBLUP}$, and $\rho_{p,w}^{GBLUP}$ that describe respectively bias, dispersion and accuracy of EBV from GBLUP;
5. Compute PEBV based on “partial data” and using PBLUP;
6. Compute statistics $\mu_{p,w}^{PBLUP}$, $b_{w,p}^{PBLUP}$, and $\rho_{p,w}^{PBLUP}$ that describe respectively bias, dispersion and accuracy of PEBV from PBLUP;
7. The statistic $\rho_{{PBLUP_{p} ,GBLUP_{p} }}$ quantifies the inverse of the relative increase in accuracy from PBLUP to GBLUP in the partial data;
8. The statistic $\rho_{{PBLUP_{w} ,GBLUP_{w} }}$ quantifies the inverse of the relative increase in accuracy from PBLUP to GBLUP in the whole data.

## Data: example using beef cattle data

### Animal population, genotypes and phenotypes

The statistics described above were tested in a real-life dataset. We used genetic and phenotypic resources (for details see Table 1) from Brahman cows (N = 995) and bulls (N = 1116) that have been widely described in the recent literature [40–42]. Yearling body weight (YWT) computed from the average of all body weights recorded between 300 and 420 days of age was used as the phenotype. The 2111 Brahman cattle were genotyped using either the Illumina BovineSNP50 BeadChip (Illumina Inc., San Diego, CA; [43]) or the BovineHD panel (Illumina Inc., San Diego, CA) that includes more than 770,000 single nucleotide polymorphisms (SNPs). Animals that were genotyped using the lower density array had their genotypes imputed to higher-density, as described previously [44]. The imputation was performed on 30 iterations of BEAGLE [45], using 519 individuals genotyped using the BovineHD chip as reference. After imputation, we retained genotypes on 729,068 SNPs, of which 651,253 were mapped to autosomal chromosomes and had a minor allele frequency (MAF) higher than 1% and were used to build the genomic relationship matrix (GRM) according to Method 1 in [46].

**Table 1:** Summary statistics for age and body weight (YWT) in yearling records used in the beef cattle data example

Sex	N	Variable	Mean	SD	Min.	Max.
Cows	995	Age (days)	361.77	12.68	323	400
		BWT (kg)	209.73	30.54	115	299
Bulls	1116	Age (days)	359.10	20.54	302	416
		BWT (kg)	243.71	29.17	138	353

### Procedure to generate partial datasets and cross-validation statistics

The data described above comprised the whole dataset. One thousand partial datasets were generated by setting a random 50% of records missing. It is worth noting that these animals are contemporaries (the resource population spans a few years and animals are not descendants from each other) and, therefore, there are no issues related to selection.

A simple breeding value mixed-model was used for the analysis of YWT with the fixed effects of contemporary group (combination of sex, year and location), age of dam at birth in year classes, and age at measurement as a covariate, and the random additive polygenic effects and residuals as random effects. Variance components estimates and BLUPs of breeding values were obtained using the Qxpak5 software [47]. All datasets were analyzed using both the pedigree-based numerator relationship matrix (NRM) and the SNP-based genomic relationship matrix (GRM).

Table 2 lists the 16 statistics that were used to compare EBV from the whole and partial datasets. Note that in order to highlight the impact of the data partitioning, some of these statistics were computed separately for the individuals in the whole and the partial datasets, in the same context as ‘reference’ and ‘validation’ individuals, respectively. For instance, $\rho_{w,p}^{v}$ is the correlation between the EBV obtained using the whole dataset and the EBV obtained using the partial dataset, but computed only by using the validation individuals that have missing phenotypes in the partial dataset, i.e. the random 50% individuals with omitted phenotypes in the ‘partial’ dataset. The EBV of those animals are predicted in the partial dataset using parent average (i.e. using pedigree) or using genomic information from relatives (i.e. using the GRM). In the whole dataset, they are predicted using own records.

**Table 2:** Set of 16 statistics used to compare predictions based on the whole and partial beef cattle datasets

Statistic	Description
$h^{2}$	REML estimate of heritability for each ‘Partial’ dataset (each random 50% missing)
$b_{w,p}$	Regression of whole on partial EBV (expectation of 1.0)
$b_{w,p}^{r}$	$b_{w,p}$ computed within reference samples (i.e. Those with phenotypes maintained in the creation of the partial sample)
$b_{w,p}^{v}$	$b_{w,p}$ computed within validation samples (i.e. Those with phenotypes treated as missing in the creation of the partial sample)
$b_{p,w}$	Regression of partial on whole EBV (expectation depends on accuracies)
$b_{p,w}^{r}$	$b_{p,w}$ computed within reference samples
$b_{p,w}^{v}$	$b_{p,w}$ computed within validation samples
$\rho_{w,p}$	Correlation between whole and partial EBV (expectation depends on accuracies)
$\rho_{w,p}^{r}$	$\rho_{w,p}$ computed within reference samples
$\rho_{w,p}^{v}$	$\rho_{w,p}$ computed within validation samples
$r\left( {y_{r} ,\hat{u}_{r} } \right)$	Correlation between the partial EBV and the adjusted phenotypes for the reference samples
$r\left( {y_{v} ,\hat{u}_{v} } \right)$	Correlation between the partial EBV and the adjusted phenotypes for the validation samples (NB. This is the conventional measure of accuracy in cross-validation genomic selection studies)
$d_{w,p}^{r}$	Difference between whole and partial EBV (in absolute value) computed within reference samples
$d_{w,p}^{v}$	Difference between whole and partial EBV (in absolute value) computed within validation samples
$Vd_{w,p}^{r}$	Variance of the difference between whole and partial EBV computed within reference samples
$Vd_{w,p}^{v}$	Variance of the difference between whole and partial EBV computed within validation samples

## Results

**Fig. 1:**
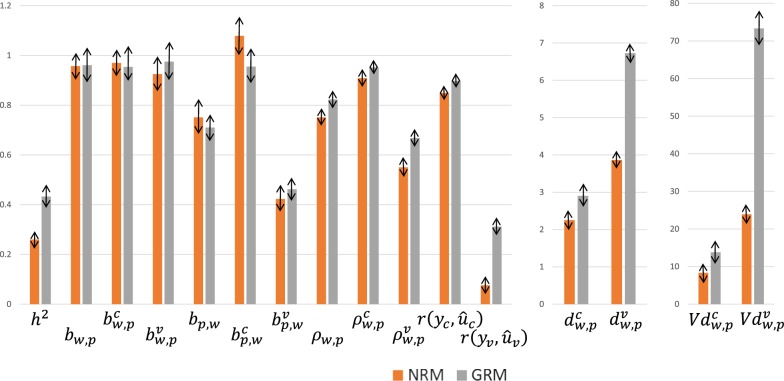
Mean value for the 16 statistics across the 1000 partial (random 50%) beef cattle datasets obtained using either the pedigree-based NRM or the SNP-based GRM. Double-ended arrows indicate ± 1 standard deviation (SD). Refer to Tables 2 and 3 for a description of the statistics and the actual values, respectively

Table 3 provides summary metrics (mean, standard deviation, minimum and maximum) for the 16 statistics across the 1000 partial datasets obtained using the NRM and the GRM. The means are also presented in the bar diagram of Fig. 1. Notable changes from using NRM versus GRM were the 66.5% increase in the estimated heritability (from 0.260 to 0.433), the 21.4% increase in $\rho_{w,p}^{v}$ (from 0.550 to 0.668) and the 4.1-fold increase in $r\left( {y_{v} ,\hat{u}_{v} } \right)$ (from 0.076 to 0.312).

**Table 3:** Summary metrics (mean, standard deviation, minimum and maximum) for the 16 statistics across the 1000 partial datasets (each one setting a random 50% as missing phenotypes) and obtained using either the pedigree-based NRM or the SNP-based GRM

Statistic	Pedigree-based NRM				SNP-based GRM			
	Mean	SD	Min.	Max.	Mean	SD	Min.	Max.
$h^{2}$	0.260	0.021	0.211	0.371	0.433	0.044	0.316	0.598
$b_{w,p}$	0.957	0.064	0.741	1.206	0.961	0.083	0.718	1.275
$b_{w,p}^{r}$	0.970	0.059	0.763	1.180	0.954	0.077	0.729	1.231
$b_{w,p}^{v}$	0.925	0.082	0.688	1.272	0.975	0.099	0.685	1.372
$b_{p,w}$	0.751	0.077	0.522	1.189	0.710	0.066	0.519	0.967
$b_{p,w}^{r}$	1.079	0.090	0.840	1.541	0.955	0.079	0.730	1.238
$b_{p,w}^{v}$	0.423	0.056	0.253	0.743	0.462	0.046	0.329	0.667
$\rho_{w,p}$	0.751	0.024	0.665	0.809	0.823	0.013	0.772	0.864
$\rho_{w,p}^{r}$	0.909	0.013	0.859	0.943	0.952	0.006	0.934	0.967
$\rho_{w,p}^{v}$	0.550	0.035	0.425	0.637	0.668	0.021	0.584	0.736
$r\left( {y_{r} ,\hat{u}_{r} } \right)$	0.849	0.012	0.804	0.892	0.898	0.015	0.852	0.944
$r\left( {y_{v} ,\hat{u}_{v} } \right)$	0.076	0.022	0.011	0.156	0.312	0.021	0.227	0.373
$d_{w,p}^{r}$	2.253	0.266	1.684	3.902	2.905	0.288	2.344	4.476
$d_{w,p}^{v}$	3.865	0.167	3.441	4.422	6.726	0.216	5.932	7.575
$Vd_{w,p}^{r}$	8.303	1.988	4.585	24.081	13.798	2.977	8.839	32.127
$Vd_{w,p}^{v}$	23.893	2.003	19.174	30.920	73.330	4.676	57.355	91.677

**Fig. 2:**
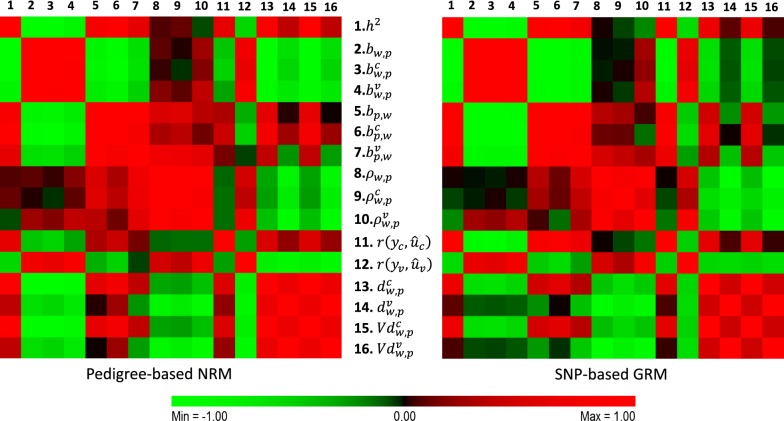
Heatmap of the correlation matrix among the 16 statistics obtained using the pedigree-based NRM (left panel) and the SNP-based GRM (right panel). Refer to Table 2 for a description of the statistics and to Supplementary Tables 1 and 2 for the actual correlation values

Figure 2 presents a heatmap of the correlation matrix among the 16 statistics obtained using the NRM and the GRM. The individual values are provided in Additional file 1: Tables S1 and S2. We observed a strong negative correlation ($r < - 0.90$ in all cases) between the heritability estimates and the regressions of EBV from whole on EBV from partial data (i.e. $b_{w,p}$, $b_{w,p}^{r}$ and $b_{w,p}^{v}$). This is consistent with the expectation of over- and under-dispersion for regression values < 1.0 and > 1.0, respectively.

One metric of interest is the correlation of EBV with precorrected phenotype (i.e., $r\left( {\varvec{y}_{new}^{*} ,\hat{\varvec{u}}_{p} } \right)$ denoted here as $r\left( {y_{v} ,\hat{u}_{v} } \right)$) since this is one of the most frequent measures of accuracy in cross-validation studies. Quite encouraging is the high correlation observed between $r\left( {y_{v} ,\hat{u}_{v} } \right)$ and the regressions of EBV from whole on EBV from partial data (i.e. $b_{w,p}$, $b_{w,p}^{r}$ and $b_{w,p}^{v}$), which ranged from 0.604 to 0.746, as well as the high positive correlation of $r\left( {y_{v} ,\hat{u}_{v} } \right)$ with the correlations between “whole’ on “partial’ (i.e. $\rho_{w,p}$, $\rho_{w,p}^{r}$ and $\rho_{w,p}^{v}$), with a maximum correlation of 0.806 between $r\left( {y_{v} ,\hat{u}_{v} } \right)$ and $\rho_{w,p}^{v}$. These results illustrate that the proposed metrics, particularly $\rho_{w,p}^{v}$, are also estimators of the accuracy of EBV based on the partial (earlier) data (termed $acc_{p}$ in our algebraical derivations).

Striking is the novel finding of the strong negative correlation of $r\left( {y_{r} ,\hat{u}_{r} } \right)$ (where $\hat{u}_{r}$ are “reference” animals in the training dataset) with $r\left( {y_{v} ,\hat{u}_{v} } \right)$. The former is bound to be high since it reflects the prediction’s goodness of fit when computed on the data that is used to build the prediction, and averaged to 0.849 and 0.898 when using the NRM and the GRM, respectively (Table 3). However, the negative correlation of $r\left( {y_{r} ,\hat{u}_{r} } \right)$ with $r\left( {y_{v} ,\hat{u}_{v} } \right)$ indicates that when the breeding value model is particularly good at fitting the reference (‘old’) data (reflected in part by a high heritability estimate), this strong fitting ability disappears when applied to the validation (‘new’) data, which seems to imply overfitting (by chance). Indeed, a very strong correlation ($r$ = 0.933) was observed between the estimate of heritability and $r\left( {y_{r} ,\hat{u}_{r} } \right)$, and a moderately strong negative correlation (*r* = − 0.543) between the estimate of heritability and $r\left( {y_{v} ,\hat{u}_{v} } \right)$ (Fig. 2 and Additional file 1: Table S2). Importantly, these problematic relationships were not observed with either $\rho_{w,p}^{r}$ or $\rho_{w,p}^{v}$.

**Fig. 3:**
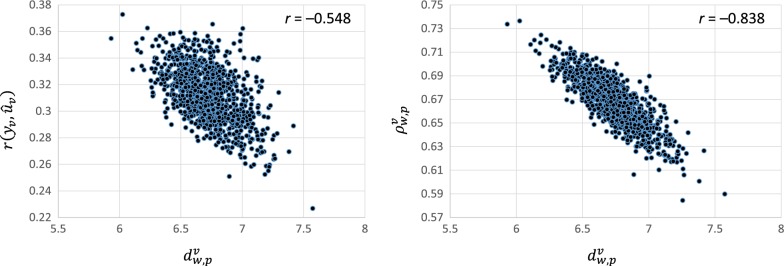
Scatter plot of the relationship of the absolute difference in EBV between the whole and partial datasets ($\varvec{d}_{{\varvec{w},\varvec{p}}}^{\varvec{v}}$) with the correlation of the EBV based on the partial data with the adjusted phenotypes ($\varvec{r}\left( {\varvec{y}_{\varvec{v}} ,\hat{\varvec{u}}_{\varvec{v}} } \right)$; left panel) and the correlation between EBV based on the whole and partial data ($\varvec{\rho}_{{\varvec{w},\varvec{p}}}^{\varvec{v}}$; right panel) across the 1000 partial (random 50%) beef cattle datasets

Finally, we explored the changes in ‘consecutive predictions’, which are represented here by the move from partial (old) to whole (new) data. We used the absolute difference between predictions (statistics $d_{w,p}^{r}$ and $d_{w,p}^{v}$) and the variance of the difference of predictions ($Vd_{w,p}^{r}$ and $Vd_{{w,{\text{p}}}}^{v}$) and explored the relationships of these with the previous 12 statistics. Please note the strong negative correlation (*r* = − 0.838) between $d_{w,p}^{v}$ and $\rho_{w,p}^{v}$. This contrasts with the not so strong correlation (*r* = − 0.548) between $d_{w,p}^{v}$ and $r\left( {y_{v} ,\hat{u}_{v} } \right)$ (Fig. 3).

Between two competing measures of accuracy, the measure that is more closely related to changes in predictions will be preferred. Based on this and our results, we conclude that $\rho_{w,p}^{v}$ is better than $r\left( {y_{v} ,\hat{u}_{v} } \right)$.

## Discussion

Thompson [13] outlined and discussed methods for the statistical validation of genetic models for genetic evaluation [14, 17, 48]. He emphasized the need for the statistical models to be based on genetic considerations. Today, different genetic considerations (e.g. oligogenic vs polygenic models) may lead to different prediction models, in particular in the area of genomic selection. Thus, the question “which model is best?” is today more important than ever. In this work, we attempt to provide quantitative geneticists with a set of tools to make their own decisions.

Why do animal breeders aim at having predictions that are unbiased in both senses, i.e. $\mu_{p} - \mu_{w} = 0$ and $b_{w,p} = 1$ ? Practically, to avoid suboptimal “biased” decisions, e.g. choosing too few or too many, or simply the wrong set of, young animals. Theoretically, best predictors, defined as conditional expectations, have optimal selection properties [49, 50], and therefore we should aim for models (not necessarily linear) that yield such best predictors. In practice, unbiasedness is a property that holds on expectation: for any real dataset, from one evaluation to the next, there will be small deviations; for instance, $\mu_{p} - \mu_{w}$ may differ from 0 just because of small noises. However, it is important to ascertain if these deviations are large (and affect the practice of selection) or not.

In selection, the expected genetic gain at the stage of selection is $\Delta G = \frac{1}{n}\varSigma \left( {EBV_{s} } \right) - \frac{1}{m}\varSigma \left( {EBV_{c} } \right) = \bar{\hat{u}}_{s} - \bar{\hat{u}}_{c}$, i.e. the average EBV of the “*s*” selected animals minus the average EBV of the “*c*” animals candidates to selection. To avoid surprises (over- or under-estimation of selected animals), we need $\bar{\hat{u}}_{s} = \bar{u}_{s}$, i.e., the estimate of the mean and the true mean of breeding values should be the same for selected animals. For this to hold, we must avoid two kinds of systematic errors: bias (wrong estimate of genetic trend) and over-/under-dispersion, which is often incorrectly referred to as “bias” in animal breeding literature. If selection is by truncation on EBV, the true mean after selection, under multivariate normality, is $\mu_{T} = \bar{\hat{u}}_{s} = \left( {1^{{\prime }} \varvec{u}} \right)/n + ir\sigma_{u}$, where $\left( {1^{{\prime }} \varvec{u}} \right)/n$ is the mean of all selection candidates and $ir\sigma_{u}$ is the genetic gain. This genetic mean is (implicitly) predicted before selection as $\mu_{E} = \left( {1^{{\prime }} \hat{\varvec{u}}} \right)/n + i\sigma_{{\hat{u}}}$. For $\mu_{T} = \mu_{E}$ to hold, we need an unbiasedness condition (i.e. $\bar{\hat{u}} = \bar{u}$ among all selection candidates) and a second condition that is $\sigma_{{\hat{u}}} = r\sigma_{u}$. The latter condition, however, only holds if $cov\left( {u,\hat{u}} \right) = var\left( {\hat{u}} \right)$, which amounts to the regression coefficient $\frac{{cov\left( {u,\hat{u}} \right)}}{{var\left( {\hat{u}} \right)}}$ to be 1. However, the equality $cov\left( {u,\hat{u}} \right) = var\left( {\hat{u}} \right)$ holds under quite restrictive conditions [30, 33]. In a frequentist context, Henderson [28, 32] proved that selection can be ignored if the model is correct, selection is contained “in the data”, and under the assumption of multivariate normality. In a Bayesian context, Sorensen et al. [34] proved that selection can be ignored if the evaluation model is correct. However, models are rarely correct, at most they are robust. In particular, the widely used animal model that includes unknown parent groups [51] is biased by construction, because genetic groups are due to genetic selection but fitted as fixed effects, which ignores established genetic theory [52].

It may be argued that for the results in [14] to hold (roughly, future errors in prediction are not correlated to current errors in prediction), future data does not need to depend on past data. This is, however, not the case if there is selection: unborn progeny of unselected animals do not yield data. In principle, models should consider selection correctly, if all information is included. A counterexample where, old data affect future errors of prediction is as follows. Consider EBV ($u_{p}$) of a young bull based on one record of the dam, with $var\left( {u_{p} } \right) = \frac{{h^{4} }}{4}$, and a subsequent EBV based on $n$ progeny records ($u_{w}$) but not on maternal performance. Then, $cov\left( {u_{p} ,u_{w} } \right) = \frac{1}{8}h^{4} \frac{2n}{n + \lambda }$, which is not equal to $var\left( {u_{p} } \right)$ because the dam performance was not included in $u_{w}$. If there is no selection, there is no problem, but this is rarely the case, and it is actually selection that creates bias due to an increase in the genetic level of the trait and a reduction in genetic variance.

Thus, we see the process of estimation of accuracy and bias of EBV by our proposed method LR as a double process. First, *checking* of the model in order to have a model that empirically has the “best” properties (estimation of bias); and then, *estimation* of its accuracy. We propose the following two-step praxis approach. First, to ascertain as best as possible that models are empirically unbiased using the statistics $\mu_{p} - \mu_{w}$ and $b_{w,p}$ which should have values 0 and 1, respectively—perhaps using, if not all, many animals (as in the original paper of Reverter et al. [14]). Second, for all models that are empirically unbiased, accuracies can be compared based on the proposed statistics, which rely on unbiasedness.

Still, there is a problem in method LR, and in all methods that rely on linear regression of “predictands” (pseudo-TBV from accurate progeny testing, less accurate EBV or precorrected records) on “predictors” (typically EBV). As shown in the “Appendix”, due to family structure and the not complete accuracy of EBV, the *true* value of the regression of TBV on EBV, i.e. the “true” $b$, has an expectation lower than 1, $E\left( b \right) < 1$. Accordingly, regression of “whole data” EBV (or of precorrected data) on “partial data” may seem to indicate bias: $E\left( {\hat{b}} \right) < 1$ *because* for the “true” $b$, $E\left( b \right) < 1$. In other words, EBV may appear to be over-dispersed when they are actually not, which holds for method LR and for any other similar method such as “predictivity” or the Interbull tests, since it is a fundamental property of the crude regression of a vector of TBV ${\mathbf{u}}$ on a vector of EBV ${\hat{\mathbf{u}}}$. It seems relevant to assess, in practice, the extent of this inequality $E\left( b \right) < 1$, since evaluations are often scaled such that $b$ (actually its estimate) is equal to 1, which implies that EBV may be too much deflated after the scaling. However, we will not address these points here, since this should be the subject of a simulation study that goes far beyond this paper. The deviation of $E\left( b \right)$ from 1 is important if the cohort, or focal group, is small and related, and it does not depend on the quality of the “predictand”. Therefore, our recommendation is to use large cohorts for validation. This bias inherent to cross-validation analysis deserves further examination in future studies.

Fixing the models to observe constraints on estimated bias should be based on rigorous genetic or statistical arguments (i.e. re-estimating variance components and heritabilities), rather than quick fixing procedures such as multiplying by constants, manipulating relationships or changing hyper-parameters of prior distributions. For instance, [53] found empirically that equaling statistics of ${\mathbf{A}}_{22}$ and ${\mathbf{G}}$ provided unbiased predictions, but this has a genetic interpretation of modelling selection and drift from the base to the genotyped population [54, 55].

In the analysis of genetic trend for litter size in pigs, Sorensen et al. [48] also emphasized “forward” cross-validation for model checking, using what we called in this paper “predictivity”, instead of relying solely on model-based predictions. Recently, Putz et al. [56] tested by simulation several methods to validate accuracies by cross-validation. They reported poor performance of comparisons of (in our notation) $\hat{u}_{w}$ and $\hat{u}_{p}$, without realizing that $r(\hat{u}_{w} ,\hat{u}_{p} )$ is not an estimator of accuracy but of ratios of accuracies. In addition, they did not simulate selection, in which case theoretical accuracy is equal to validation accuracies.

We have shown that precorrection of phenotypes using whole data may bias the result of predictivity. This is particularly relevant for small contemporary groups such as in dairy or beef cattle as opposed to, say, sheep or aquaculture species. Some measure of error in precorrection due to estimation of contemporary groups should be reported in cross-validation results. Although the ranking of methods should be similar, estimates of population accuracies may be biased. Comparing $\hat{u}_{w}$ and $\hat{u}_{p}$, as we propose in this work, might be a better option, although it involves more parametric assumptions.

One final consideration involves discussing the difference between population and individual accuracy. Quoting [18]: “For response to selection, the [population] accuracy should reflect the correlation between true and EBV in the candidates for selection, which is a property of a population, not of an individual. For the stability of EBV, the accuracy should reflect the standard error of an EBV, which can be defined for a single individual.” Our work deals with population accuracies, not with individual accuracies. The former are useful for model selection and for genetic gain; the latter are useful for individual decisions. The population accuracy is not a function of individual accuracies. For instance, consider full sibs that are evaluated by using parent average and for which their parents are known exactly: individual accuracy is 0.71. However, population accuracy is 0, since all full-sibs have exactly the same parent average. Thus, population accuracies involve both individual reliabilities and co-reliabilities [24, 25].

## Conclusions

In this paper, we present properties of cross-validation measures obtained from successive genetic evaluations. These measures allow estimation of population accuracies and biases, which are of interest to quantitative geneticists in general, and animal and plant breeders in particular. We hope that with these tools, researchers can report and compare competing prediction models, in particular for complex cases such as for lowly heritable traits or for indirect genetic values such as maternal effects.

## Additional file

**Additional file 1: Tables S1 and S2.** Correlation among the 16 statistics employed in the cross-validation study of the beef cattle dataset using the pedigree-based NRM or the SNP-based GRM (NB: These are the values used to generate the left and right panels of Fig. 2

## Appendix

In this Appendix, we quantify the possible systematic error in $E\left( {\frac{X}{Y}} \right) \approx \frac{E\left( X \right)}{E\left( Y \right)}$, where X and Y are successive EBV, or EBV and TBV. Here, we show that this systematic error is small if the number of EBV in X and Y is large (in the hundreds or thousands). The second-order approximation of $E\left( {\frac{X}{Y}} \right)$ is $E\left( {\frac{X}{Y}} \right) \approx \frac{E\left( X \right)}{E\left( Y \right)} - \frac{{Cov\left( {X,Y} \right)}}{{E\left( Y \right)^{2} }}$ [57]. Consider for instance, $b_{w,p} = \frac{{cov\left( {{\hat{\mathbf{u}}}_{p} ,{\hat{\mathbf{u}}}_{w} } \right)}}{{var\left( {{\hat{\mathbf{u}}}_{p} } \right)}} = \frac{{\frac{1}{n}\left( {{\hat{\mathbf{u}}}_{p} - {\bar{\hat{\mathbf{u}}}}_{p} } \right)^{'} \left( {{\hat{\mathbf{u}}}_{w} - {\bar{\hat{\mathbf{u}}}}_{w} } \right)}}{{\frac{1}{n}\left( {{\hat{\mathbf{u}}}_{p} - {\bar{\hat{\mathbf{u}}}}_{p} } \right)^{'} \left( {{\hat{\mathbf{u}}}_{p} - {\bar{\hat{\mathbf{u}}}}_{p} } \right)}}$. The systematic error incurred in the approximation $E\left( {\frac{X}{Y}} \right) \approx \frac{E\left( X \right)}{E\left( Y \right)}$ is $- \frac{{Cov\left( {X,Y} \right)}}{{E\left( Y \right)^{2} }}$, where $X = \frac{1}{n}\left( {{\hat{\mathbf{u}}}_{p} - {\bar{\hat{\mathbf{u}}}}_{p} } \right)^{'} \left( {{\hat{\mathbf{u}}}_{w} - {\bar{\hat{\mathbf{u}}}}_{w} } \right) = \frac{1}{n}\left( {{\hat{\mathbf{u}}}_{p} {\mathbf{S}{\hat{\mathbf{u}}}}_{w} } \right)$ where ${\mathbf{S}} = {\mathbf{I}} - \frac{1}{n}{\mathbf{J}}$ and $Y = \frac{1}{n}\left( {{\hat{\mathbf{u}}}_{p} - {\bar{\hat{\mathbf{u}}}}_{p} } \right)^{{\prime }} \left( {{\hat{\mathbf{u}}}_{p} - {\bar{\hat{\mathbf{u}}}}_{p} } \right) = \frac{1}{n}\left( {{\hat{\mathbf{u}}}_{p} {\mathbf{S}{\hat{\mathbf{u}}}}_{p} } \right)$. To simplify notation, consider ${\mathbf{K}} = {\mathbf{G}} - {\mathbf{C}}_{p}^{uu}$, ${\mathbf{K}} = \left\{ {k_{ij} } \right\}$. Thus,

$$E\left( Y \right)^{2} = \left( {\frac{1}{n}tr\left( {{\mathbf{SK}}} \right)} \right)^{2} = \left( {\overline{{diag\left( {\mathbf{K}} \right)}} - {\bar{\mathbf{K}}}} \right)^{2} .$$

The expression for the covariance of bilinear forms is $Cov\left( {{\mathbf{x}}_{1}^{{\prime }} {\mathbf{A}}_{12} {\mathbf{x}}_{2} ,{\mathbf{x}}_{3} {\mathbf{A}}_{34} {\mathbf{x}}_{4} } \right) = tr\left( {{\mathbf{A}}_{12} {\mathbf{C}}_{23} {\mathbf{A}}_{34} {\mathbf{C}}_{41} + {\mathbf{A}}_{12} {\mathbf{C}}_{24} {\mathbf{A}}_{43} {\mathbf{C}}_{31} } \right) + {\varvec{\upmu}}_{1}^{{\prime }} {\mathbf{A}}_{12} {\mathbf{C}}_{23} {\mathbf{A}}_{34} {\varvec{\upmu}}_{4} + {\varvec{\upmu}}_{1}^{{\prime }} {\mathbf{A}}_{12} {\mathbf{C}}_{24} {\mathbf{A}}_{43} {\varvec{\upmu}}_{3} + {\varvec{\upmu}}_{2}^{'} {\mathbf{A}}_{21} {\mathbf{C}}_{13} {\mathbf{A}}_{34} {\varvec{\upmu}}_{4} + {\varvec{\upmu}}_{2}^{{\prime }} {\mathbf{A}}_{21} {\mathbf{C}}_{14} {\mathbf{A}}_{43} {\varvec{\upmu}}_{3}$ (${\mathbf{C}}$ is the covariance matrix across $\varvec{x}_{i}$; p 58 [30]). Applied to our case, $Cov\left( {{\mathbf{x}}_{1}^{{\prime }} {\mathbf{A}}_{12} {\mathbf{x}}_{2} ,{\mathbf{x}}_{3} {\mathbf{A}}_{34} {\mathbf{x}}_{4} } \right) = Cov\left( {{\hat{\mathbf{u}}}_{p} {\mathbf{S}{\hat{\mathbf{u}}}}_{w} ,{\hat{\mathbf{u}}}_{p} {\mathbf{S}{\hat{\mathbf{u}}}}_{p} } \right)$, this yields (as $Cov\left( {\hat{\mathbf{u}}_{p} ,\hat{\mathbf{u}}_{w} } \right) = Cov\left( {\hat{\mathbf{u}}_{p} ,\hat{\mathbf{u}}_{p} } \right) = {\mathbf{K}}$):

$$Cov\left( {X,Y} \right) = \frac{2}{{n^{2} }}tr\left( {{\mathbf{SKSK}}} \right).$$

The terms linked to the means disappear, as before, because they have the form $\mu {\mathbf{1}}^{{\prime }} {\mathbf{S}}^{{\prime }} {\mathbf{KS}}{\mathbf{1}}\mu$ which has a value of 0. The expression $Cov\left( {X,Y} \right) = \frac{2}{{n^{2} }}tr\left( {{\mathbf{SKSK}}} \right)$ can be computed explicitly for ideal cases. A slightly more enlightening expression is, after algebra,

$$Cov\left( {X,Y} \right) = 2\overline{{var\left( {{\mathbf{k}}_{i,:} } \right)}} - 2var\left( {{\bar{\mathbf{k}}}_{i,:} } \right),$$

twice the average variance within rows minus the variance of rows means of ${\mathbf{K}} = {\mathbf{G}} - {\mathbf{C}}_{p}^{uu}$. Putting all together results in:

$$\frac{{Cov\left( {X,Y} \right)}}{{E\left( Y \right)^{2} }} = \frac{{2\overline{{var\left( {{\mathbf{k}}_{i,:} } \right)}} - 2var\left( {{\bar{\mathbf{k}}}_{i,:} } \right)}}{{\overline{{diag\left( {\mathbf{K}} \right)}} - {\bar{\mathbf{K}}}}}.$$

This is always positive, which means that the $b$ estimated as the linear regression of ${\hat{\mathbf{u}}}_{w}$, but also the “true” $b$ of the regression of true EBV ${\mathbf{u}}$ on ${\hat{\mathbf{u}}}_{p}$, has an expectation less than 1, *even when the model is correct*, contrary to common assertions. The expectation of $b$ is actually $1 - \frac{{2\overline{{var\left( {{\mathbf{k}}_{i,:} } \right)}} - 2var\left( {{\bar{\mathbf{k}}}_{i,:} } \right)}}{{\overline{{diag\left( {\mathbf{K}} \right)}} - {\bar{\mathbf{K}}}}}$. When the value of $\frac{{2\overline{{var\left( {{\mathbf{k}}_{i,:} } \right)}} - 2var\left( {{\bar{\mathbf{k}}}_{i,:} } \right)}}{{\overline{{diag\left( {\mathbf{K}} \right)}} - {\bar{\mathbf{K}}}}}$ is high (i.e. sufficiently larger than 0), a punctual estimate of $\hat{b}_{w,p} = \frac{{cov\left( {{\hat{\mathbf{u}}}_{p} ,{\hat{\mathbf{u}}}_{w} } \right)}}{{var\left( {{\hat{\mathbf{u}}}_{p} } \right)}}$ with value equal to 1 means that the estimators $\hat{u}_{p}$ are deflated—too much regressed. This raises questions on the use of cross-validation to choose the best model for evaluation. The underestimation depends on the total number of individuals in the focal set, on their relationships (on ${\mathbf{G}}$) and the accuracies and co-reliabilities on the “partial” dataset (on ${\mathbf{C}}_{p}^{uu}$) but it does not depend on the final reliabilities on ${\mathbf{C}}_{w}^{uu}$ (which implies that the derivation applies for TBV). Inclusion of sibs increases systematic error. For instance, $n = 100$ with half-sibs of size 10 and information in “partial” evaluation equal to 1 observation with $h^{2} = 0.3$, results in $E\left( b \right) = 0.94$. Increasing to $n = 400$ results in $E\left( b \right) = 0.98$. Setting $n = 100$ with families of five half-sibs results on $E\left( b \right) = 0.96$. These systematic errors deserve further exploration (e.g. properties of the estimators for different accuracies and family structures)—but this is out of the scope of this paper.
