# Correction to: Semi-parametric estimates of population accuracy and bias of predictions of breeding values and future phenotypes using the LR method

**DOI:** 10.1186/s12711-019-0511-5

**Published:** 2019-11-22

**Authors:** Andres Legarra, Antonio Reverter

**Affiliations:** 1 INRA, UMR1388 GenPhySE, 31326 Castanet-Tolosan, France; 2 CSIRO Agriculture and Food, 306 Carmody Rd., St. Lucia, QLD 4067 Australia

## Correction to: Genet Sel Evol (2018) 50:53 10.1186/s12711-018-0426-6

After publication of original article [1], the authors noticed that there was an error.

In the section “Quadratic forms of estimated breeding values” we wrote, based on Taylor series approximations, that:

- “In the remainder of this paper, we assume that the expectation of a ratio of quadratic forms is equal to the ratio of the expectations. The “Appendix” shows that this holds when the number of individuals included in the statistics is large (several hundred or more) or when they are not structured into very large sibships. Otherwise, as shown in the “Appendix”, both the true regression coefficient $b = cov\left( {{\hat{\mathbf{u}}}_{p} ,{\mathbf{u}}} \right)/var\left( {{\hat{\mathbf{u}}}_{p} } \right)$  and its estimator $\hat{b} = cov\left( {{\hat{\mathbf{u}}}_{p} ,{\hat{\mathbf{u}}}_{w} } \right)/var\left( {{\hat{\mathbf{u}}}_{p} } \right)$ have an expectation less than 1, even when the model is perfect and the EBV have the right dispersion.”

This statement is wrong, because a further expansion of the Taylor series (until its 3rd term) shows that the expectation of both $b$ and $\hat{b}$ is 1. The correct paragraph should be:

- “In the remainder of this paper, we assume that the expectation of a ratio of quadratic forms is equal to the ratio of the expectations. The “Correction” below shows that this holds (up to the second order of the Taylor series expansion) when the BLUP properties hold, as assumed throughout the paper.”

Moreover, the Abstract reads “Contrary to common belief, the regression of true on estimated breeding values is (on expectation) lower than 1 for small or related validation sets, due to family structures.” whereas it should read “Up to the second term of a Taylor series expansion, the regression of true on estimated breeding values is (on expectation) equal to 1, even for small or related validation sets.”

Further proof (equivalent to the Appendix) is below. According to [2] the second-order Taylor series expansion of a ratio is $E\left( {\frac{X}{Y}} \right) \approx \frac{E\left( X \right)}{E\left( Y \right)} - \frac{{Cov\left( {X,Y} \right)}}{{E\left( Y \right)^{2} }} + \frac{Var\left( Y \right)E\left( X \right)}{{E\left( Y \right)^{3} }}$. Consider the bias in the estimation of the ratio $b = \frac{{cov\left( {{\hat{\mathbf{u}}}_{p} ,{\mathbf{u}}} \right)}}{{var\left( {{\hat{\mathbf{u}}}_{p} } \right)}}$ using the estimator $\hat{b} = \frac{{cov\left( {{\hat{\mathbf{u}}}_{p} ,{\hat{\mathbf{u}}}_{w} } \right)}}{{var\left( {{\hat{\mathbf{u}}}_{p} } \right)}}$. In our case:

$$\hat{b}_{{w,p}}  = \frac{{cov\left( {\widehat{{\mathbf{u}}}_{p} ,\widehat{{\mathbf{u}}}_{w} } \right)}}{{var\left( {\widehat{{\mathbf{u}}}_{p} } \right)}} = {\text{ }}\frac{{\frac{1}{n}\left( {\widehat{{\mathbf{u}}}_{p}  - \overline{{\widehat{{\mathbf{u}}}}} _{p} } \right)^{\prime } \left( {\widehat{{\mathbf{u}}}_{w}  - \overline{{\widehat{{\mathbf{u}}}}} _{w} } \right)}}{{\frac{1}{n}\left( {\widehat{{\mathbf{u}}}_{p}  - \overline{{\widehat{{\mathbf{u}}}}} _{p} } \right)^{\prime } \left( {\widehat{{\mathbf{u}}}_{p}  - \overline{{\widehat{{\mathbf{u}}}}} _{p} } \right)}}. $$

Therefore, we expand $E\left( {\frac{X}{Y}} \right)$ for $= \frac{1}{n}\left( {\widehat{{\mathbf{u}}}_{p}  - \overline{{\widehat{{\mathbf{u}}}}} _{p} } \right)^{\prime } \left( {\widehat{{\mathbf{u}}}_{w}  - \overline{{\widehat{{\mathbf{u}}}}} _{w} } \right) = \frac{1}{n}\left( {\widehat{{\mathbf{u}}}^{\prime } _{p} {\mathbf{S^{\prime}S\widehat{u}}}_{w} {\text{ }}} \right) = \frac{1}{n}\left( {\widehat{{\mathbf{u}}}_{p} {{\mathbf{S\widehat{{u}}}}_{w}} } \right),$ where ${\mathbf{S}} = {\mathbf{I}} - \frac{1}{n}{\mathbf{J}}$ and $Y = \frac{1}{n}\left( {\widehat{{\mathbf{u}}}_{p}  - \overline{{\widehat{{\mathbf{u}}}}} _{p} } \right)^{\prime } \left( {\widehat{{\mathbf{u}}}_{p}  - \widehat{{\mathbf{u}}}_{p} } \right) = \frac{1}{n}\left( {\widehat{{\mathbf{u}}}_{p} {\mathbf{S\widehat{u}}}_{p} } \right).$ The expression for the covariance of bilinear forms (under normality) is:

$$ \begin{aligned}& Cov\left( {{\mathbf{x}}_{1}^{\varvec{\prime}} {\mathbf{A}}_{12} {\mathbf{x}}_{2} ,{\mathbf{x}}_{3} {\mathbf{A}}_{34} {\mathbf{x}}_{4} } \right) \\ & \quad  = tr\left( {{\mathbf{A}}_{12} {\mathbf{C}}_{23} {\mathbf{A}}_{34} {\mathbf{C}}_{41} + {\mathbf{A}}_{12} {\mathbf{C}}_{24} {\mathbf{A}}_{43} {\mathbf{C}}_{31} } \right) + {\varvec{\upmu}}_{1}^{\prime} {\mathbf{A}}_{12} {\mathbf{C}}_{23} {\mathbf{A}}_{34} {\varvec{\upmu}}_{4} \\ & \qquad + {\varvec{\upmu}}_{1}^{'} {\mathbf{A}}_{12} {\mathbf{C}}_{24} {\mathbf{A}}_{43} {\varvec{\upmu}}_{3} + {\varvec{\upmu}}_{2}^{\prime} {\mathbf{A}}_{21} {\mathbf{C}}_{13} {\mathbf{A}}_{34} {\varvec{\upmu}}_{4} + {\varvec{\upmu}}_{2}^{'} {\mathbf{A}}_{21} {\mathbf{C}}_{14} {\mathbf{A}}_{43} {\varvec{\upmu}}_{3} \end{aligned}, $$

(${\mathbf{C}}$ is the covariance matrix across ${\mathbf{x}}_{i}$; chapter 2 Equation 58 in [3]). Applied to our case, this yields:

$$ Cov\left( {X,Y} \right) = \frac{2}{{n^{2} }}tr\left( {{\mathbf{S}}\left( {{\mathbf{G}} - {\mathbf{C}}_{p}^{uu} } \right){\mathbf{S}}\left( {{\mathbf{G}} - {\mathbf{C}}_{p}^{uu} } \right)} \right).  $$

The terms linked to the means disappear, as before, because they have the form $\mu \mathbf{1}^{\prime}{\mathbf{S^{\prime}}}\left( {{\mathbf{G}} - {\mathbf{C}}_{p}^{uu} } \right){\mathbf{S1}}\mu$, which has a value of 0. Then, we have:

$$ \begin{gathered}   E\left( Y \right) = E\left( {\frac{1}{n}\left( {\widehat{{\mathbf{u}}}_{p}  - \overline{{\widehat{{\mathbf{u}}}}} _{p} } \right)^{\prime } \left( {\widehat{{\mathbf{u}}}_{p}  - \overline{{\widehat{{\mathbf{u}}}}} _{p} } \right)} \right) = \frac{1}{n}tr\left( {{\mathbf{S}}\left( {{\mathbf{G}} - {\mathbf{C}}_{p}^{{uu}} } \right)} \right), \hfill \\   Var\left( Y \right) = Cov\left( {Y,Y} \right) = \frac{2}{{n^{2} }}tr\left( {{\mathbf{S}}\left( {{\mathbf{G}} - {\mathbf{C}}_{p}^{{uu}} } \right){\mathbf{S}}\left( {{\mathbf{G}} - {\mathbf{C}}_{p}^{{uu}} } \right)} \right), \hfill \\   E\left( X \right){\text{ }} = E\left( {\frac{1}{n}\left( {\widehat{{\mathbf{u}}}_{p}  - \overline{{\widehat{{\mathbf{u}}}}} _{p} } \right)^{\prime } \left( {\widehat{{\mathbf{u}}}_{w}  - \overline{{\widehat{{\mathbf{u}}}}} _{w} } \right)} \right) = \frac{1}{n}tr\left( {{\mathbf{S}}\left( {{\mathbf{G}} - {\mathbf{C}}_{p}^{{uu}} } \right)} \right), \hfill \\  \end{gathered}  $$

which are all the elements needed. To simplify notation, consider ${\mathbf{K}} = {\mathbf{G}} - {\mathbf{C}}_{p}^{uu}$. Putting all together gives:

$$ \begin{aligned} & - \frac{{Cov\left( {X,Y} \right)}}{{E\left( Y \right)^{2} }} + \frac{Var\left( Y \right)E\left( X \right)}{{E\left( Y \right)^{3} }}  \\ &= - \frac{{\frac{2}{{n^{2} }}tr\left( {{\mathbf{SKSK}}} \right)}}{{\left( {\frac{1}{n}tr\left( {{\mathbf{SK}}} \right)} \right)^{2} }} + \frac{{\frac{2}{{n^{2} }}tr\left( {{\mathbf{SKSK}}} \right)\frac{1}{n}tr\left( {{\mathbf{SK}}} \right)}}{{\left( {\frac{1}{n}tr\left( {{\mathbf{SK}}} \right)} \right)^{3} }} \\ & = - \frac{{2tr\left( {{\mathbf{SKSK}}} \right)}}{{\left( {tr\left( {{\mathbf{SK}}} \right)} \right)^{2} }} + \frac{{2tr\left( {{\mathbf{SKSK}}} \right)tr\left( {{\mathbf{SK}}} \right)}}{{\left( {tr\left( {{\mathbf{SK}}} \right)} \right)^{3} }}  \end{aligned}$$

which cancels out. Therefore, up to the 3rd order approximation, the bias in the estimation of $b = \frac{{cov\left( {{\hat{\mathbf{u}}}_{p} ,{\mathbf{u}}} \right)}}{{var\left( {{\hat{\mathbf{u}}}_{p} } \right)}}$ using the estimator $\hat{b} = \frac{{cov\left( {{\hat{\mathbf{u}}}_{p} ,{\hat{\mathbf{u}}}_{w} } \right)}}{{var\left( {{\hat{\mathbf{u}}}_{p} } \right)}}$ is 0. In addition, this shows that the expected value of $b = \frac{{cov\left( {{\hat{\mathbf{u}}}_{p} ,{\mathbf{u}}} \right)}}{{var\left( {{\hat{\mathbf{u}}}_{p} } \right)}}$ is also 1 since the same derivation as above holds with ${\mathbf{u}}$ in the place of ${\mathbf{u}}_{w}$.
