# Supplementary material for: Semi-parametric estimates of population accuracy and bias of predictions of breeding values and future phenotypes using the LR method
Source: Genet Sel Evol. 2018 Nov 6;50:53. doi: 10.1186/s12711-018-0426-6 (PMC6219059; doi:10.1186/s12711-018-0426-6)
Supplement: Supplementary file 1 — Additional file 1: Tables S1 and S2. Correlation among the 16 statistics employed in the cross-validation study of the beef cattle dataset using the pedigree-based NRM or the SNP-based GRM (NB: These are the values used to generate the left and right panels of Fig. 2 [file 12711_2018_426_MOESM1_ESM.docx]

**Supplementary Table 1.** Correlation among the 16 statistics employed in the cross-validation study of the beef cattle dataset using the pedigree-based NRM (NB: These are the values used to generate the left panel of Figure 2).

|  | 1 | 2 | 3 | 4 | 5 | 6 | 7 | 8 | 9 | 10 | 11 | 12 | 13 | 14 | 15 | 16 |
| --- | --- | --- | --- | --- | --- | --- | --- | --- | --- | --- | --- | --- | --- | --- | --- | --- |
| 1.$h^{2}$ | 1.000 | -0.914 | -0.957 | -0.783 | 0.821 | 0.937 | 0.622 | 0.068 | 0.109 | -0.061 | 0.632 | -0.518 | 0.886 | 0.385 | 0.887 | 0.386 |
| 2.$b_{w,p}$ | -0.914 | 1.000 | 0.986 | 0.959 | -0.782 | -0.904 | -0.574 | 0.087 | 0.015 | 0.253 | -0.420 | 0.729 | -0.911 | -0.574 | -0.902 | -0.572 |
| 3.$b_{w,p}^{c}$ | -0.957 | 0.986 | 1.000 | 0.898 | -0.806 | -0.933 | -0.603 | 0.033 | -0.025 | 0.171 | -0.495 | 0.653 | -0.914 | -0.506 | -0.904 | -0.504 |
| 4.$b_{w,p}^{v}$ | -0.783 | 0.959 | 0.898 | 1.000 | -0.687 | -0.795 | -0.477 | 0.180 | 0.089 | 0.389 | -0.276 | 0.820 | -0.850 | -0.654 | -0.842 | -0.654 |
| 5.$b_{p,w}$ | 0.821 | -0.782 | -0.806 | -0.687 | 1.000 | 0.951 | 0.932 | 0.543 | 0.567 | 0.356 | 0.305 | -0.314 | 0.614 | 0.019 | 0.622 | 0.001 |
| 6.$b_{p,w}^{c}$ | 0.937 | -0.904 | -0.933 | -0.795 | 0.951 | 1.000 | 0.806 | 0.297 | 0.367 | 0.127 | 0.431 | -0.477 | 0.778 | 0.250 | 0.783 | 0.242 |
| 7.$b_{p,w}^{v}$ | 0.622 | -0.574 | -0.603 | -0.477 | 0.932 | 0.806 | 1.000 | 0.717 | 0.698 | 0.616 | 0.138 | -0.046 | 0.378 | -0.227 | 0.391 | -0.253 |
| 8.$\rho_{w,p}$ | 0.068 | 0.087 | 0.033 | 0.180 | 0.543 | 0.297 | 0.717 | 1.000 | 0.945 | 0.920 | -0.107 | 0.488 | -0.283 | -0.774 | -0.270 | -0.804 |
| 9.$\rho_{w,p}^{c}$ | 0.109 | 0.015 | -0.025 | 0.089 | 0.567 | 0.367 | 0.698 | 0.945 | 1.000 | 0.821 | -0.127 | 0.374 | -0.262 | -0.670 | -0.248 | -0.692 |
| 10.$\rho_{w,p}^{v}$ | -0.061 | 0.253 | 0.171 | 0.389 | 0.356 | 0.127 | 0.616 | 0.920 | 0.821 | 1.000 | -0.123 | 0.693 | -0.393 | -0.848 | -0.379 | -0.876 |
| 11.  $r\left( y_{c},\hat{u}_{c} \right)$ | 0.632 | -0.420 | -0.495 | -0.276 | 0.305 | 0.431 | 0.138 | -0.107 | -0.127 | -0.123 | 1.000 | -0.259 | 0.541 | 0.211 | 0.539 | 0.225 |
| 12. $r\left( y_{v},\hat{u}_{v} \right)$ | -0.518 | 0.729 | 0.653 | 0.820 | -0.314 | -0.477 | -0.046 | 0.488 | 0.374 | 0.693 | -0.259 | 1.000 | -0.720 | -0.806 | -0.714 | -0.818 |
| 13. $d_{w,p}^{c}$ | 0.886 | -0.911 | -0.914 | -0.850 | 0.614 | 0.778 | 0.378 | -0.283 | -0.262 | -0.393 | 0.541 | -0.720 | 1.000 | 0.684 | 0.986 | 0.677 |
| 14. $d_{w,p}^{v}$ | 0.385 | -0.574 | -0.506 | -0.654 | 0.019 | 0.250 | -0.227 | -0.774 | -0.670 | -0.848 | 0.211 | -0.806 | 0.684 | 1.000 | 0.671 | 0.975 |
| 15. ${Vd}_{w,p}^{c}$ | 0.887 | -0.902 | -0.904 | -0.842 | 0.622 | 0.783 | 0.391 | -0.270 | -0.248 | -0.379 | 0.539 | -0.714 | 0.986 | 0.671 | 1.000 | 0.679 |
| 16. ${Vd}_{w,p}^{v}$ | 0.386 | -0.572 | -0.504 | -0.654 | 0.001 | 0.242 | -0.253 | -0.804 | -0.692 | -0.876 | 0.225 | -0.818 | 0.677 | 0.975 | 0.679 | 1.000 |

**Supplementary Table 2.** Correlation among the 16 statistics employed in the cross-validation study of the beef cattle dataset using the SNP-based GRM (NB: These are the values used to generate the right panel of Figure 2).

|  | 1 | 2 | 3 | 4 | 5 | 6 | 7 | 8 | 9 | 10 | 11 | 12 | 13 | 14 | 15 | 16 |
| --- | --- | --- | --- | --- | --- | --- | --- | --- | --- | --- | --- | --- | --- | --- | --- | --- |
| 1.$h^{2}$ | 1.000 | -0.948 | -0.973 | -0.864 | 0.905 | 0.971 | 0.776 | 0.005 | -0.050 | -0.188 | 0.933 | -0.543 | 0.673 | 0.090 | 0.681 | 0.062 |
| 2.$b_{w,p}$ | -0.948 | 1.000 | 0.990 | 0.972 | -0.932 | -0.975 | -0.820 | -0.003 | -0.008 | 0.262 | -0.853 | 0.668 | -0.574 | -0.084 | -0.578 | -0.060 |
| 3.$b_{w,p}^{c}$ | -0.973 | 0.990 | 1.000 | 0.929 | -0.928 | -0.985 | -0.810 | -0.009 | 0.011 | 0.214 | -0.890 | 0.604 | -0.594 | -0.075 | -0.598 | -0.050 |
| 4.$b_{w,p}^{v}$ | -0.864 | 0.972 | 0.929 | 1.000 | -0.897 | -0.915 | -0.802 | 0.009 | -0.039 | 0.330 | -0.751 | 0.746 | -0.516 | -0.094 | -0.520 | -0.075 |
| 5.$b_{p,w}$ | 0.905 | -0.932 | -0.928 | -0.897 | 1.000 | 0.965 | 0.952 | 0.341 | 0.277 | 0.049 | 0.808 | -0.449 | 0.475 | -0.204 | 0.494 | -0.237 |
| 6.$b_{p,w}^{c}$ | 0.971 | -0.975 | -0.985 | -0.915 | 0.965 | 1.000 | 0.862 | 0.117 | 0.106 | -0.128 | 0.881 | -0.551 | 0.590 | 0.002 | 0.603 | -0.024 |
| 7.$b_{p,w}^{v}$ | 0.776 | -0.820 | -0.810 | -0.802 | 0.952 | 0.862 | 1.000 | 0.512 | 0.403 | 0.284 | 0.685 | -0.256 | 0.328 | -0.393 | 0.351 | -0.430 |
| 8.$\rho_{w,p}$ | 0.005 | -0.003 | -0.009 | 0.009 | 0.341 | 0.117 | 0.512 | 1.000 | 0.841 | 0.854 | 0.001 | 0.500 | -0.412 | -0.896 | -0.379 | -0.934 |
| 9.$\rho_{w,p}^{c}$ | -0.050 | -0.008 | 0.011 | -0.039 | 0.277 | 0.106 | 0.403 | 0.841 | 1.000 | 0.618 | -0.054 | 0.304 | -0.566 | -0.720 | -0.533 | -0.743 |
| 10.$\rho_{w,p}^{v}$ | -0.188 | 0.262 | 0.214 | 0.330 | 0.049 | -0.128 | 0.284 | 0.854 | 0.618 | 1.000 | -0.142 | 0.806 | -0.440 | -0.838 | -0.414 | -0.868 |
| 11.  $r\left( y_{c},\hat{u}_{c} \right)$ | 0.933 | -0.853 | -0.890 | -0.751 | 0.808 | 0.881 | 0.685 | 0.001 | -0.054 | -0.142 | 1.000 | -0.465 | 0.593 | 0.060 | 0.596 | 0.032 |
| 12. $r\left( y_{v},\hat{u}_{v} \right)$ | -0.543 | 0.668 | 0.604 | 0.746 | -0.449 | -0.551 | -0.256 | 0.500 | 0.304 | 0.806 | -0.465 | 1.000 | -0.510 | -0.548 | -0.497 | -0.558 |
| 13. $d_{w,p}^{c}$ | 0.673 | -0.574 | -0.594 | -0.516 | 0.475 | 0.590 | 0.328 | -0.412 | -0.566 | -0.440 | 0.593 | -0.510 | 1.000 | 0.477 | 0.990 | 0.472 |
| 14. $d_{w,p}^{v}$ | 0.090 | -0.084 | -0.075 | -0.094 | -0.204 | 0.002 | -0.393 | -0.896 | -0.720 | -0.838 | 0.060 | -0.548 | 0.477 | 1.000 | 0.450 | 0.960 |
| 15. ${Vd}_{w,p}^{c}$ | 0.681 | -0.578 | -0.598 | -0.520 | 0.494 | 0.603 | 0.351 | -0.379 | -0.533 | -0.414 | 0.596 | -0.497 | 0.990 | 0.450 | 1.000 | 0.450 |
| 16. ${Vd}_{w,p}^{v}$ | 0.062 | -0.060 | -0.050 | -0.075 | -0.237 | -0.024 | -0.430 | -0.934 | -0.743 | -0.868 | 0.032 | -0.558 | 0.472 | 0.960 | 0.450 | 1.000 |
